# Receptor tyrosine kinase inhibitors negatively impact on pro-reparative characteristics of human cardiac progenitor cells

**DOI:** 10.1038/s41598-022-13203-3

**Published:** 2022-06-16

**Authors:** Andrew J. Smith, Prashant Ruchaya, Robert Walmsley, Kathleen E. Wright, Fiona C. Lewis-McDougall, Jacquelyn Bond, Georgina M. Ellison-Hughes

**Affiliations:** 1 School of Biomedical Sciences, Faculty of Biological Sciences, University of Leeds, Woodhouse Lane, Leeds, LS2 9JT UK; 2 Centre for Human and Applied Physiological Sciences & Centre for Stem Cells and Regenerative Medicine, School of Basic and Medical Biosciences, Faculty of Life Sciences & Medicine, Guy’s Campus, King’s College London, London, SE1 1UL UK; 3 School of Health, Sport and Biosciences, College of Applied Health and Communities, University of East London, Arthur Edwards Building, Room 5.29, Stratford Campus, Water Ln, London, E15 4LZ UK; 4 William Harvey Research Institute, Barts and the London School of Medicine, Queen Mary University of London, London, EC1M 6BQ UK; 5 Leeds Institute of Medical Research, Faculty of Medicine and Health, University of Leeds, St. James’s Hospital, Beckett Street, Leeds, LS9 7TF UK; 6 School of Biomedical Sciences, Faculty of Biological Sciences, University of Leeds, Room 7.52b, Garstang Building, Woodhouse Lane, Leeds, LS2 9JT UK

**Keywords:** Cell death, Cell signalling

## Abstract

Receptor tyrosine kinase inhibitors improve cancer survival but their cardiotoxicity requires investigation. We investigated these inhibitors’ effects on human cardiac progenitor cells in vitro and rat heart in vivo. We applied imatinib, sunitinib or sorafenib to human cardiac progenitor cells, assessing cell viability, proliferation, stemness, differentiation, growth factor production and second messengers. Alongside, sunitinib effects were assessed in vivo. Inhibitors decreased (*p* < 0.05) cell viability, at levels equivalent to ‘peak’ (24 h; imatinib: 91.5 ± 0.9%; sunitinib: 83.9 ± 1.8%; sorafenib: 75.0 ± 1.6%) and ‘trough’ (7 days; imatinib: 62.3 ± 6.2%; sunitinib: 86.2 ± 3.5%) clinical plasma levels, compared to control (100% viability). Reduced (*p* < 0.05) cell cycle activity was seen with imatinib (29.3 ± 4.3% cells in S/G2/M-phases; 50.3 ± 5.1% in control). Expression of PECAM-1, Nkx2.5, Wnt2, linked with cell differentiation, were decreased (*p* < 0.05) 2, 2 and 6-fold, respectively. Expression of HGF, p38 and Akt1 in cells was reduced (*p* < 0.05) by sunitinib. Second messenger (p38 and Akt1) blockade affected progenitor cell phenotype, reducing c-kit and growth factor (HGF, EGF) expression. Sunitinib for 9 days (40 mg/kg, i.p.) in adult rats reduced (*p* < 0.05) cardiac ejection fraction (68 ± 2% *vs*. baseline (83 ± 1%) and control (84 ± 4%)) and reduced progenitor cell numbers. Receptor tyrosine kinase inhibitors reduce cardiac progenitor cell survival, proliferation, differentiation and reparative growth factor expression.

## Introduction

The development of receptor tyrosine kinase inhibitors (RTKIs) as anti-cancer therapies was a significant advance in the field of oncotherapy. These treatments, targeted at specific cancer types, lead to improved survival with reduced side-effects compared to earlier anti-cancer drug treatments^1,2^. Target cancers for RTKIs were initially primarily haematological malignancies, specifically chronic myeloid leukaemia, but as further RTKIs were developed, the range of cancer types RTKIs were applied to increased substantially^3,4^. Consequently, the population undergoing RTKI treatment has risen steadily year on year.

While RTKIs feature less severe toxic side-effect profiles than many older anti-cancer drugs, these side-effects are still an important complication of RTKI therapy. A principal concern is cardiotoxicity, occurring in sizable sub-populations of patients using RTKIs: 8% of patients treated with imatinib mesylate (IM)^5^, 18% of patients treated with sorafenib tosylate (ST)^6^ and 28% of patients treated with sunitinib malate (SM)^7^. While this compares favourably with the cardiotoxic effects seen in up to 36% of patients using doxorubicin^6^, it is nonetheless a significant side-effect in a substantial percentage of patients. As patient survival has increased with RTKI use, this has led to an expanding population of patients suffering from effects of RTKI cardiotoxicity in the long term^2^. Considering the relative risk of RTKIs inducing cardiotoxicity, it is notable that a trial adding ST to the existing drug regimen of acute myeloid leukaemia patients (daunorubicin and cytarabine) increased the risk ratio for cardiac events to 3.46^8^.

RTKI-induced cardiotoxicity has manifested clinically as a significant reduction of left ventricular ejection fraction (LVEF) following IM treatment^9^. Reduced LVEF was also seen in 28% of patients after SM treatment, with LVEF reduced by ≥ 15% in 19% of patients and 28% of patients also developing hypertension^7^. Meta-analysis of VEGFR-blocking RTKIs (SM, ST, pazopanib) identified a 1.5% risk of a fatal adverse event, with cardiac complications being the second-commonest cause for this^10^. The mechanisms underlying acute RTKI cardiotoxicity are principally effects on cardiomyocytes, with IM inducing endoplasmic reticular stress, loss of mitochondrial membrane potential and subsequent cardiomyocyte death^9^. Similarly, SM induces cardiomyocyte death via mitochondrial stress and energy depletion^11^, whereas ST causes cardiomyocyte hypertrophy in vivo and cardiomyocyte death at doses equivalent to clinical peak plasma levels^12^. Recent work has also shown negative RTKI impacts on cardiac fibroblasts^13^.

The discovery of a rare population of cardiac stem/progenitor cells (CPCs) resident in the adult myocardium^14^, including human^15^, identified a potential cell source for cardiac repair and regeneration. These cells can be isolated from the heart and possess the defining stem/progenitor cell characteristics, being self-renewing, clonogenic and multipotent; differentiating into cardiac cell types in vitro^14–18^ and in vivo^14,19–22^. Their discovery was complemented by evidence of new cardiomyocyte formation throughout adult life in humans, albeit importantly this was only to a limited extent, 0.5–1% per annum^23^. The extent to which these rare stem/progenitor cells generate new cardiomyocytes in situ is however highly debated^22,24,25^, particularly in the context of myocardial infarction, where several studies have shown limited-to-no new cardiomyocyte formation^26–28^.

Separate from cardiomyocyte formation however, the ability of CPCs to support cardiac repair via generation and secretion of pro-survival and pro-reparative growth factors is widely-recognised^29–31^. Further, effective CPC-driven regeneration has been seen after diffuse myocardial injury^21^, an injury which significantly contrasts with myocardial infarction due to the patent coronary circulation and largely intact myocardial tissue architecture. While an interesting report shows that a key mechanism of cell therapy post-infarction is stimulation of the innate immune response within the heart, induced by any cells including dead cells^32^, this does not explain the effect of CPCs seen following diffuse injury, as recovery was not seen with infusion of fibroblasts^21,33^.

Considering their role in repair following diffuse damage with a low (~ 10%) dropout of cardiomyocytes^21,25,34^, the possibility arises that a component of cardiotoxic diffuse damage could be the loss or incapacity of the CPC population. Supporting this is the finding that exposure to doxorubicin, an anthracycline chemotherapeutic, in early life depletes the CPC population and increases cardiac vulnerability to injury in later life^35^. In addition, it has been shown that doxorubicin can induce CPC senescence in situ, or cause DNA damage, cell cycle arrest, cell death and reduce cell migration in vitro^36^. Furthermore, cardiac functional loss from doxorubicin cardiotoxicity was significantly reduced by application of ex vivo expanded CPCs^37^.

Our hypothesis is that RTKIs with known clinical cardiotoxicity are toxic to CPCs, thereby reducing cardiac capacity for in situ repair. To determine this, we examined effects of three clinically-used RTKIs on human CPC survival, proliferation, expression of pro-survival and regenerative growth factors and ability to generate differentiated progeny. In addition, we confirmed whether the RTKI most lethal to human CPCs in vitro could also affect CPC numbers in vivo and assessed any impacts on cardiac function alongside this. The RTKIs used in this study were selected based on three criteria: (1) widely-used clinically; (2) known cardiotoxic effects; (3) acting on distinct combinations of RTKs (IM: c-kit, Abl, PDGFRα; SM: VEGFR1/2/3, PDGFRs; ST: VEGFR2/3, PDGFRs) shown to be expressed by CPCs^18^.

## Methods

### Cell isolation and culture

Samples of human myocardium were obtained from spare tissue discarded during cardiac surgery, with all tissue donors giving informed written consent prior to their inclusion in the study, in accordance with the principles of the Declaration of Helsinki (NREC approved study number 08/H1306/91). The tissue sample was thoroughly chopped and then underwent sequential digestions using type 2 collagenase (Lorne Laboratories). The cardiac small cell fractions were separated from the cardiomyocytes and undigested tissue through a 40 µm mesh, then pooled and centrifuged. Magnetic-assisted cell sorting technology (Miltenyi) was used to first eliminate the CD45^pos^ haematopoietic lineage cells and CD31^pos^ endothelial (progenitor) cells. The c-kit^pos^ fraction was purified from the CD45^neg^ and CD31^neg^ population by direct labelling using magnetically-tagged antibodies (Miltenyi)^15^. Cells were maintained at or below 80% confluency in CPC growth medium^15^ on plasticware coated with CellStart (Thermo Fisher).

### RTKI treatments in vitro

Receptor tyrosine kinase inhibitors used were imatinib mesylate (IM), sunitinib malate (SM), sorafenib tosylate (ST), prepared as concentrated stock solutions (IM and SM in water, ST in DMSO) and stored at −20 °C for further use. Drugs were applied by preparing final concentrations in CPC growth medium^15^ and administering to cells in vitro. The concentrations of RTKIs used in this study were selected based on data for the levels seen in clinical plasma samples at peak^38,39^ and trough^40,41^ concentrations, with higher and lower concentrations to identify any dose–response patterns (100 µM being above the levels seen clinically). Concentrations used in this study were respectively peak: 10 µM IM, 1.25 µM SM, 10 µM ST and trough: 5 µM IM, 0.5 µM SM, 5 µM ST. The two dose types (higher concentrations for shorter periods, lower for longer) were used as at the study outset it was not known whether drug concentration or exposure duration would be more critical, nor if this would vary across the different CPC characteristics studied.

### In vivo methods

Male Wistar rats aged approximately 11 weeks (~ 250 g weight) were used, with all experimental procedures carried out in accordance with the regulations of the British Home Office Animals (Scientific Procedures) Act of 1986. Study approval was obtained from the King’s College London Animal Welfare and Ethical Review Body, the U.K. Home Office and all procedures conformed to the guidelines from Directive 2010/63/EU of the European Parliament. Animals were in groups of n = 6, with each experimental unit being an individual animal. Sample size estimation was based on prior work: to identify 10% reduction in ejection fraction, assuming a SD of 6%, with α value 0.05, a sample of six gave a power of 0.80. No exclusion criteria or randomisation were used. Animals received once-daily intraperitoneal (i.p.) injections of 40 mg/kg body weight SM (Cayman Chemical) dissolved in PBS, with an equivalent volume of PBS alone given to control group animals, for 9 days. Echocardiography was performed by an experienced operator under light general anaesthesia (1.5% isoflurane and 0.5 ml/min O_2_) using a Vevo 770 Imaging System (Visual Sonics), to determine cardiac diameters and cardiac functional outputs. During anaesthesia, heart rates were maintained between 250 and 300 bpm, in order to ensure comparable data across individuals. To determine recovery or non-recovery, the criteria used were that rats with ejection fraction that had returned to within two standard deviations of baseline ejection fraction were classed as having recovered. This work was performed at the Rayne Institute, St. Thomas’ Hospital campus, King’s College London. Rats were singly caged and housed in holding rooms with regulated 12 h light/dark cycle maintained at 22 °C ± 1 °C with a 40%-60% humidity; acclimatisation was for 7 days on arrival to the facility. Rat chow diet and water were provided ad libitum*.* Animals were sacrificed by euthanasia using cervical dislocation (schedule 1 procedure) at the conclusion of their involvement in the study. The work carried out in this study is compliant with the ARRIVE guidelines.

### Tissue analysis

Hearts were fixed in 10% formalin for 24 h, followed by an overnight dehydration in 70% ethanol. Hearts were sectioned into apex, mid and base, and processed for paraffin embedding. 10 µm thick slices of heart from the apex, mid and base regions were cut using a Reichert-Jung, 1140/autocut microtome and slide mounted for direct immunohistochemical fluorescent staining. Sections were deparaffinised with Histoclear, prior to rehydration and antigen retrieval (boiling in citrate buffer for 30 min). Immunofluorescent antibody labelling (c-kit, R&D; α-sarcomeric actin, Sigma; AlexaFluor-488 and -568 secondary antibodies, Thermo Fisher) proceeded according to standard methods, with DAPI nuclear staining (Sigma) and coverslip mounting with Slowfade (Life Technologies). For collagen staining, slides were rinsed with Histoclear twice, prior to rehydration and staining with Sirius Red for 60 min, followed by 0.01 M HCl wash to remove excess stain. The slides were dehydrated and mounted in DPX prior to viewing. Slides were viewed using a Zeiss LSM 700 confocal microscope, and image analysis (cell counts or area calculations) performed using ImageJ, with 18 random fields of view analysed per subject (six individual subjects per group).

### Viability assay

Cell viability was analysed using a fluorescein diacetate assay, in which non-fluorescent esterified fluorescein (fluorescein diacetate, Sigma) is applied to living cells, which take up and cleave it to produce intracellular fluorescein. Medium was removed and cells incubated with a freshly-made solution of DMEM containing 5 µg/ml FDA for 10 min at 37 °C^42^. After this, fluorescence was determined (excitation 485 nm; emission 538 nm) with a Vari-skan plate reader (Thermo Fisher), with viability expressed relative to untreated control cells. Each assay was carried out in triplicate, with independent cell preparations used for each assay repeat.

### Cell cycle flow cytometry

Cell cycle activity was assessed by DNA labelling with 250 µg/ml propidium iodide, applied with 0.8% Triton and 800 µg/ml RNase type 1a (all Sigma) in PBS for 10 min at 20 °C. Following this, cells were analysed using a FACSAria II flow cytometer (BD Biosciences), with signal detected on a linear fluorescence scale (excitation: 561 nm; emission: 610 ± 20 nm). Cells were quantified according to: single or double DNA complement (corresponding to G0/G1 or G2/M phases respectively), and intermediate (corresponding to S-phase).

### Differentiation and quantitative analysis of immunostaining

Cells were placed in differentiation medium for 14 days, using the conditions and media described previously for respective cardiac cell type lineages^18^. At the end of this process, cells were fixed with 4% paraformaldehyde for 20 min on ice, then washed in PBS prior to immunostaining. Cells were stained with primary antibodies overnight: alpha-sarcomeric actin (Sigma), von Willebrand factor (Dako) or smooth muscle actin (Sigma), before staining with a corresponding AlexaFluor-488 secondary antibody (Thermo Fisher) for 1 h at 37 °C. Cells were then stained with TOTO to delineate the cytoplasm and DAPI to mark nuclei, then transferred to an Operetta plate reader (Perkin Elmer) for visualisation. Data were analysed and each separate iteration (four for each differentiation condition, against each RTKI) objectively quantified using Columbus software (Perkin Elmer).

### RNA isolation and real-time quantitative PCR

RNA was isolated using the RNeasy Mini kit (Qiagen), with the eluted total RNA concentration and purity assessed using a Nanodrop spectrophotometer (Thermo Fisher). This isolated RNA was used as a template for cDNA synthesis with the iScript Reverse Transcription kit (Bio-Rad). This cDNA was used for SYBR Green-based real-time qPCR analysis (Bio-Rad), with thermal cycling and real-time PCR detection achieved using a CFX96 Touch system (Bio-Rad). Primers were designed using Primer-BLAST (NIH), manufactured (Sigma) and used at a final concentration of 300 nM. Each result was obtained from three independent biological repeats: RNA isolated after experiment and samples run in triplicate for technical repeats.

### Immunocytochemistry

Cells were spun onto pre-coated glass slides (Fisher) using a CytoSpin centrifuge (Thermo Fisher), before fixation by CellFixx (Thermo Fisher) and storage for later immunocytochemistry using standard methods; antibodies used: Ki67 (Thermo Fisher) and BrdU (Roche). Slides were viewed and representative confocal microscope images obtained (Zeiss LSM 710 and LSM software).

### Western blotting

Western blotting was performed using protein samples from cells lysed in RIPA buffer and denatured at 95 °C, before loading in SDS-PAGE gels, in 8 or 12% polyacrylamide gels according to target protein size. Samples then underwent electrophoresis at 50 V for 10 min followed by 100 V for 90 min, prior to transfer onto nitrocellulose or PVDF membranes (Bio-Rad). After this, membranes were washed with 5% milk solution to block non-specific binding (or 5% BSA for phosphoproteins) and immunolabelled with relevant primary antibodies at 4 °C overnight. After secondary immunolabelling with HRP-conjugated antibodies (at room temperature for 1 h), membranes were immersed in Supersignal West Pico Plus 1:1 chemiluminescent solution (Thermo Fisher) and viewed using a G:Box Chemi-XT4, with images analysed using GENESys and ImageJ software. Blots of ‘housekeeping’ protein β-actin were used for corrected quantification, with the corrected quantified values presented graphically below the blots. Western blotting was carried out to verify that gene expression findings were associated with impacts on protein expression: the blots were quantified relative to their loading controls so that the differences in blots could be clearly assessed.

### Statistical analysis

Statistical analyses were performed by Student’s *t*-test or ANOVA with Holm-Šídák multiple comparisons test as appropriate (after data passed the Shapiro–Wilk normality test), using the GraphPad Prism 7 software. Data are presented as mean ± SEM, with a *p* value of < 0.05 considered to be statistically significant.

## Results

### RTKIs reduce CPC survival in vitro

**Figure 1:**
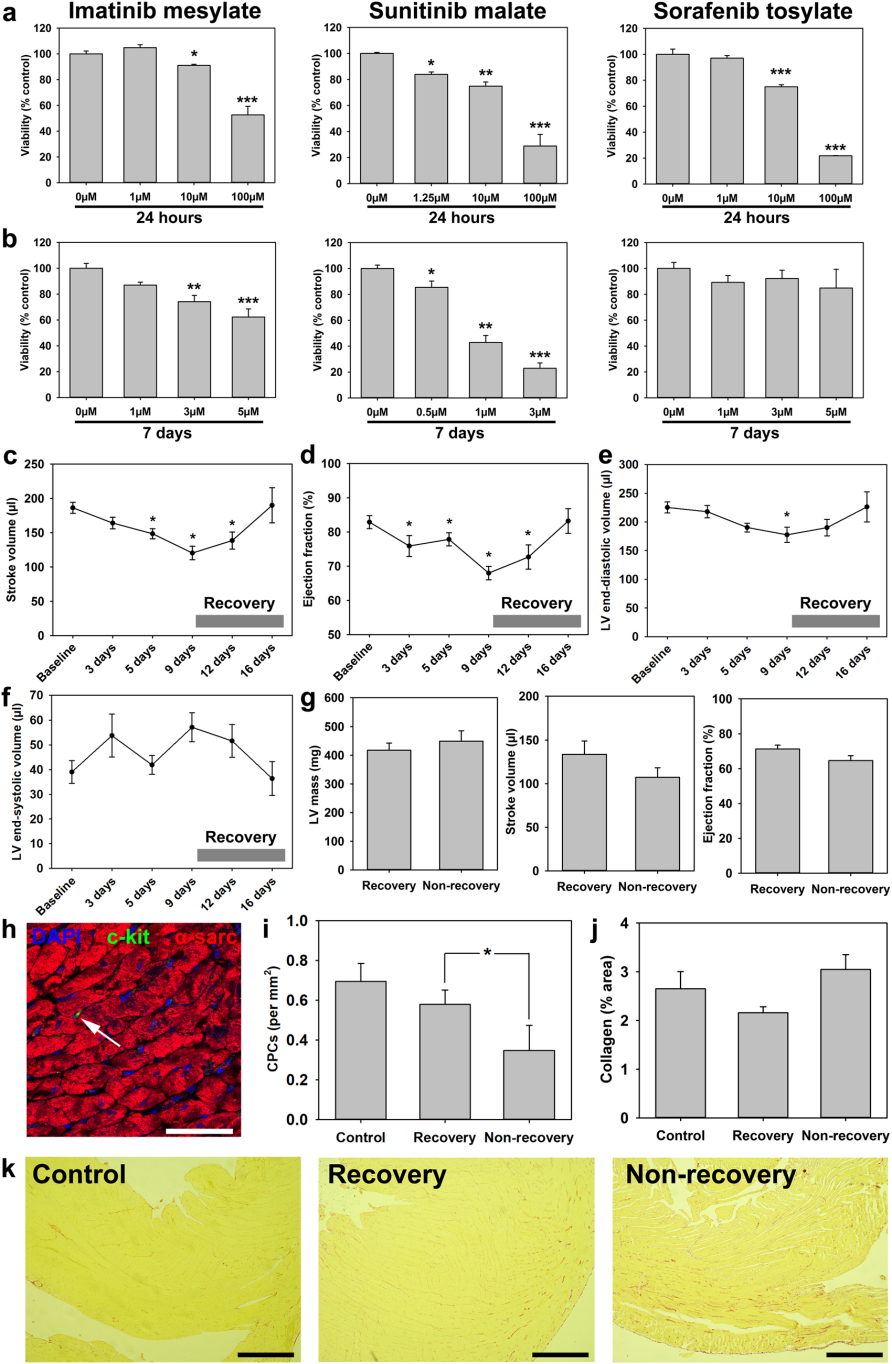
RTKIs reduce CPC survival in vitro and impact upon cardiac function in vivo. RTKIs impact negatively on human CPC viability following (**a**) 24-h exposures to 1–100 µM IM, 1.25–100 µM SM or 1–100 µM ST (n = 4) and (**b**) 7-day exposures to 1–5 µM IM, 0.5–3 µM SM or 1–5 µM ST (n = 4). Application of SM to adult male Wistar rats in vivo caused (**c**) a reduction of cardiac stroke volume after 9 days (n = 12), with (**d**) similar impact on ejection fraction (n = 12), followed by recovery of both 7 days after cessation of SM. Left ventricular end-diastolic volume (**e**) was transiently reduced, but LV end-systolic volume (**f**) was not altered (n = 12). (**g**) Echocardiography analysis of LV mass, stroke volume and ejection fraction in recovery and non-recovery groups after 9 days’ SM treatment (n = 6). (**h**) Representative image of immunostaining: α-sarcomeric actin (red), c-kit (green), DAPI (blue) showing CPC in situ (arrow; scale bar = 50 µm). **(i)** Immunohistochemical analysis of c-kit^pos^ cell number (per mm^2^) in control, recovery and non-recovery groups (n = 6). (**j**) Histochemical analysis of cardiac fibrosis in control, recovery and non-recovery groups (n = 6), with (**k**) representative images of Sirius red collagen staining (scale bar = 750 µm). All data are mean ± SEM; significance was determined by Student’s *t*-test for (**i**), or ANOVA with Holm-Šídák post hoc test to identify differences in all other comparisons (*p < 0.05; **p < 0.01; ***p < 0.001).

Our first objective was to determine the impact of RTKIs on the viability of human CPCs in vitro. When treated with RTKIs at concentrations equivalent to ‘peak’ plasma concentrations for 24 h, all 3 RTKIs, IM, SM and ST decreased viability in a dose-dependent manner, with 100 µM being the most potent (*p* < 0.05 in each case) (Fig. 1a). Further examination of lower doses (equivalent to ‘trough’ plasma concentrations) treated for 7 days showed decreased viability with increasing dose of IM and SM, however trough levels of ST did not significantly affect CPC viability (Fig. 1b). Therefore, RTKIs had significant detrimental effects on human CPC viability, at clinically-comparable doses, with morphological changes seen consistent with this (Supplementary Fig. 1a). No impact on CPC viability was seen using a DMSO-only control for ST experiments (data not shown).

### Sunitinib malate impairs cardiac function in vivo

Due to the potent peak and trough effects of SM on CPC viability in vitro, we tested the effect of SM administration on CPCs in vivo, to affirm this toxicity was reproduced for CPCs in situ, while also assessing cardiac function. Daily injection of 40 mg/kg SM caused a significant (*p* < 0.05) reduction in stroke volume (SV) by 5 days, from 186.3 ± 8.1 µl to 148.3 ± 7.4 µl (Fig. 1c). In addition, ejection fraction (EF) was significantly (*p* < 0.05) reduced after 3 days, reaching a minimum of 67.9 ± 1.9% at 9 days, compared to a baseline of 82.9 ± 1.9% (Fig. 1d). There was a transient reduction in left ventricular end-diastolic volume (Fig. 1e) but not left ventricular end-systolic volume (Fig. 1f). However, upon cessation of drug treatment at 9 days, there was recovery in SV (Fig. 1c) and EF (Fig. 1d) by 7 days in the overall population. Similarly, left ventricular internal dimension and posterior wall thickness showed reductions that resolved on treatment cessation, with no significant change in left ventricular anterior wall thickness (Supplementary Figs. 1b–d).

This initially suggested that SM cardiotoxicity did not lead to persistent reduction in cardiac function, but analysis of responses in individual subjects indicated a more complex possible interpretation. When responses were separated according to individual animal physiological responses, a sub-population was seen in which reduced body weight did not recover after ceasing drug administration (Supplementary Fig. 1e). When cardiac ejection fractions of these rats were individually plotted, two distinct patterns were seen; SM cessation followed by recovery of EF in 50% of subjects (subjects 13, 19, 20, 21, 23, 24) or SM cessation followed by non-recovery of EF (subjects 14, 15, 16, 17, 18, 22), some of which died prematurely (Supplementary Fig. 1f).

To determine if the CPC toxicity seen in vitro was replicated by sunitinib toxicity in vivo, we analysed CPC numbers histologically, complemented by analysis of myocardial fibrosis. Due to the already-identified ejection fraction findings, these histological analyses and further echocardiography (LV mass, cardiac function) were performed contrasting data from animals which recovered with those which did not (including those dying prematurely). Recovery was defined as animals with final cardiac ejection fractions readings with no significant difference to the start-of-study baseline (within two standard deviations of baseline). Echocardiographic analysis at completion of SM treatment (9 days) identified no discrepancy in severity of drug impact on cardiac function at this point, with no significant differences in LV mass, stroke volume or ejection fraction between recovery and non-recovery groups (Fig. 1g). Analysis of CPC numbers in situ (Fig. 1h) showed significantly fewer (*p* = 0.043) in the non-recovery group compared to the recovery group (221.8 ± 92.1 *vs.* 427.0 ± 56.4, per 10^6^ cardiomyocytes) (Fig. 1i). Staining of CD45 and CD31 alongside c-kit excluded the possibility that the cells identified in situ were mast cells or endothelial cells (Supplementary Fig. 1g) and this reduction in CPCs was also evident relative to cardiomyocyte number (Supplementary Fig. 1h). Examination of myocardial fibrosis by collagen staining identified no significant differences between control, recovery and non-recovery groups (Fig. 1j,k). Therefore, RTKIs have a detrimental impact on CPC survival in vitro, and when administered in vivo reduce CPC numbers and cause transient impairment in cardiac function. In animals that do not show recovery of cardiac function following cessation of RTKI administration, CPC numbers in the myocardium were significantly reduced.

### RTKIs impair CPC proliferation

**Figure 2:**
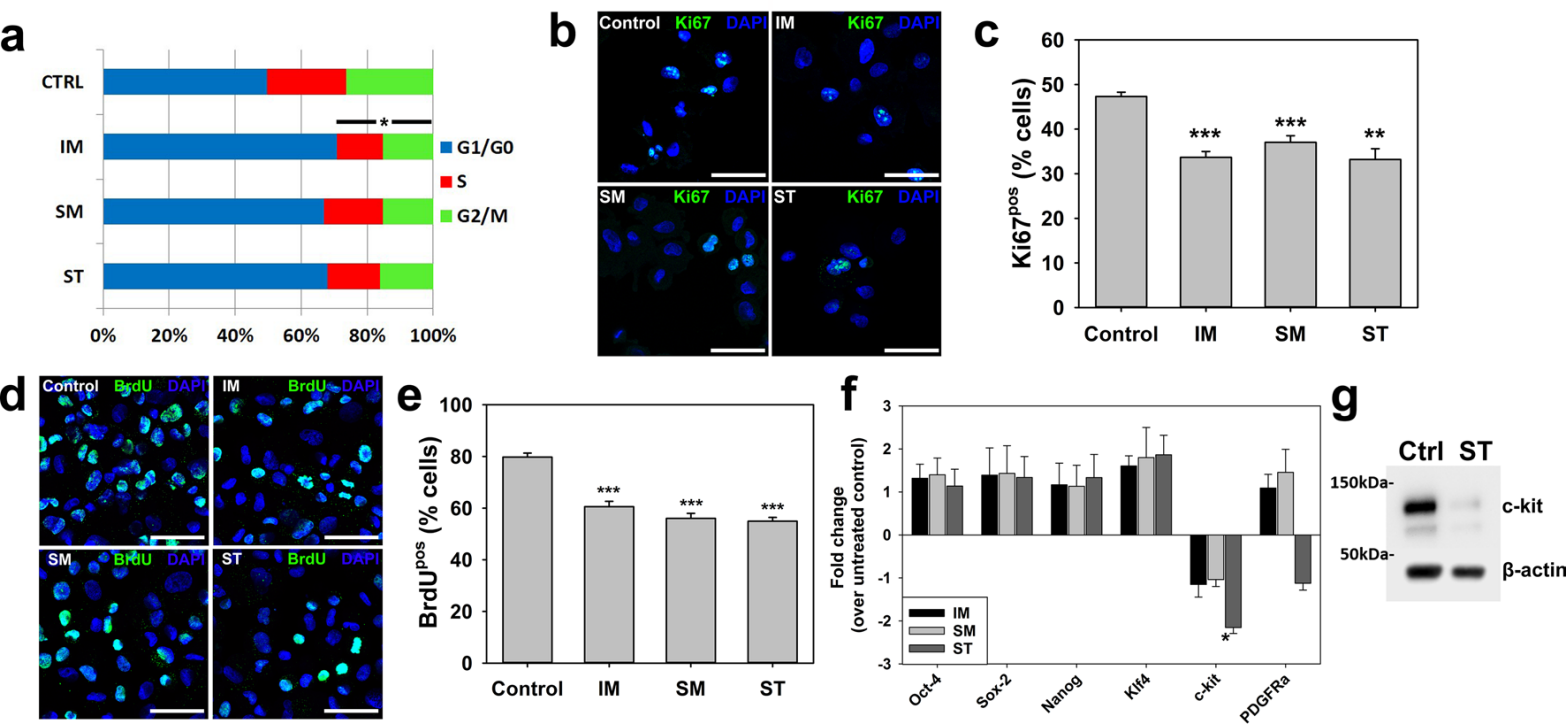
CPC population maintenance reduced by RTKIs. (**a**) CPC cell cycle activity was reduced by RTKIs: 10 µM IM, 1.25 µM SM or 10 µM ST for 24 h reduced cells in S, G2 and M phases (n = 3). (**b**) Representative Ki67 ICC images (scale bar = 50 µm). (**c**) Ki67 protein expression reduced relative to control CPCs by 10 µM IM, 1.25 µM SM or 10 µM ST (n = 3). (**d**) Representative BrdU ICC images (scale bar = 50 µm). (**e**) CPC proliferation reduced by RTKIs: percentage BrdU^pos^ CPCs reduced by 10 µM IM, 1.25 µM SM or 10 µM ST over 48 h (n = 3). (**f**) Real-time qPCR analysis of CPC ‘stemness’ and CPC marker gene expression: reduction in c-kit expression by 5 µM ST after 7 days (n = 3). (**g**) Downregulation of c-kit protein expression by 5 µM ST after 7 days. All data are mean ± SEM; significance was determined by ANOVA with Holm-Šídák post hoc test to identify differences (**p < 0.01; ***p < 0.001). Blots cropped to relevant data, with optical density normalized to the loading control protein; full blot images presented in Supplementary Fig. 6.

We next focused on RTKI impact on CPC cell cycle activity. Application of peak levels of all 3 RTKIs for 24 h impacted negatively on the cell cycle activity of CPCs, with the number of cells in S/G2/M phases reduced from 50.3 ± 5.1% in control cells to 29.3 ± 4.3% with 10 µM IM; 33.1 ± 8.5% with 1.25 µM SM; and 32.1 ± 10.9% with 10 µM ST (Fig. 2a). These data were supported by counts of viable cells after RTKI treatments, using both Trypan blue and propidium iodide staining methods to exclude dead cells: (Supplementary Fig. 2a–e). These findings were further affirmed by quantification of CPCs expressing Ki67 over a 24 h period, with CPCs expressing Ki67 reduced from 47.3 ± 0.9% Ki67^pos^ in control conditions to 33.7 ± 1.3% by 10 µM IM, 37.0 ± 1.5% with 1.25 µM SM and 33.2 ± 2.4% with 10 µM ST (Fig. 2b,c). Further assessment used BrdU labelling to measure DNA synthesis, and showed reduced numbers of BrdU^pos^ CPCs following peak levels of RTKIs (Fig. 2d,e). Therefore, RTKIs decreased CPC proliferation.

We determined RTKI impact on expression of the ‘stemness’ genes linked with cell multipotency (Oct-4, Sox-2, Nanog, Klf-4) and CPC surface receptor markers, c-kit and PDGFRα. Application of each RTKI for 7 days at ‘trough’ concentrations did not significantly impact on expression of pluripotency-linked genes (Fig. 2f). All three RTKIs showed a down regulation of c-kit expression in CPCs, compared to untreated control CPCs (Fig. 2g), however only ST was found to cause significant (*p* < 0.001) change in gene expression and reduce protein expression (Fig. 2f,g). ST exposure also caused onefold down-regulation of PDGFRα expression in CPCs, compared to untreated control CPCs. Application of ‘peak’ doses for 24 h did not cause any significant changes (Supplementary Fig. 2f). Furthermore, we confirmed via flow cytometry the presence of the RTKs VEGFR1, VEGFR2 and VEGFR3 in human CPCs, although PDGFRα or β expression was minimal (Supplementary Fig. 3). Finally, we examined the impact of RTKIs on PDGFR and VEGFR expression using flow cytometry, finding that RTKIs did not significantly impact on the expression of VEGFRs or PDGFRs (Supplementary Fig. 4). Only a slight reduction in PDGFRα following ST was seen, consistent with the non-significant reduction in PDGFRα expression already seen in our PCR data, and minor fluctuations in PDGFRβ expression (Fig. 2f). Therefore, RTKI exposure decreased expression of c-kit, which is one of the key surface receptors of CPCs.

### RTKIs reduce CPC differentiation potential

**Figure 3:**
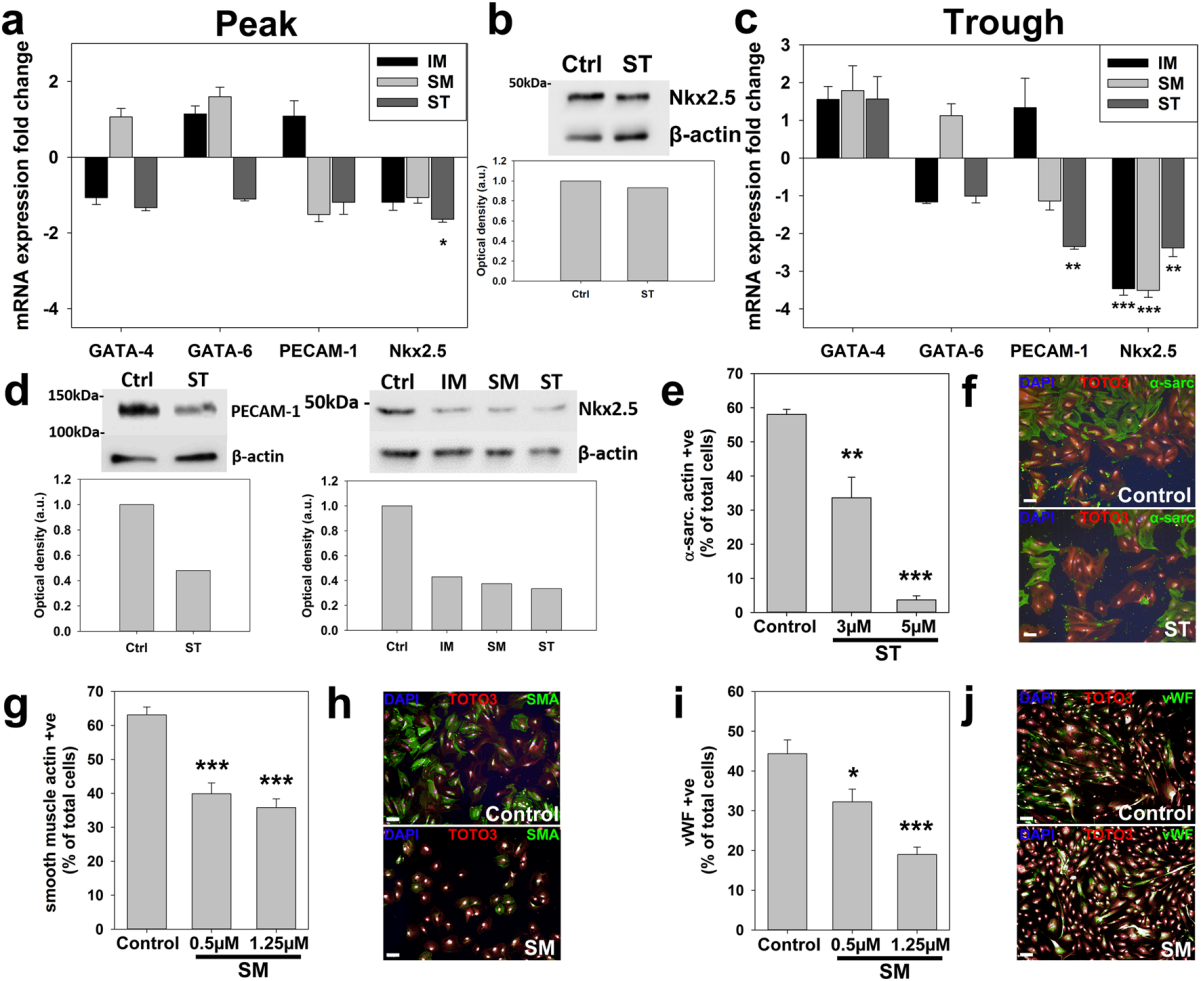
RTKIs reduce CPC potential to differentiate. (**a**) Real-time qPCR analysis of CPC early differentiation markers: Nkx2.5 reduced by 10 µM ST for 24 h and (**b**) but without changes to Nkx2.5 protein expression in CPCs post ST treatment. (**c**) Real-time qPCR analysis of CPC early differentiation: PECAM-1 reduced by 5 µM ST and Nkx2.5 reduced by 5 µM IM, 0.5 µM SM or 5 µM ST at 7 days; (**d**) protein expression was also assessed. Directed differentiation of CPCs in vitro determined that: (**e**) percentage of CPCs expressing α-sarcomeric actin was reduced by ST (n = 4), (**f**) representative images (scale bar = 100 µm); (**g**) percentage of CPCs expressing smooth muscle actin was reduced by SM (n = 4), (**h**) representative images (scale bar = 100 µm); (**i**) percentage of CPCs expressing von Willebrand factor was reduced by SM (n = 4), (**j**) representative images (scale bar = 100 µm). All data are mean ± SEM; significance was determined by ANOVA with Holm-Šídák post hoc test to identify differences (*p < 0.05; **p < 0.01; ***p < 0.001). Blots cropped to relevant data, with optical density normalized to the loading control protein; full blot images presented in Supplementary Fig. 6.

We next examined the effects of RTKIs on the potential of CPCs to differentiate into cardiomyocyte-like, endothelial and smooth muscle cell lineages^14,18,21^, by examining impacts on undifferentiated CPCs and on differentiation. Exposure to peak levels of RTKIs over 24 h reduced Nkx2.5 mRNA expression (Fig. 3a), with ST decreasing (*p* = 0.027) Nkx2.5 expression at gene but not protein level (Fig. 3a,b). After 7-day exposure to trough levels, Nkx2.5 expression was reduced (*p* < 0.01) between 2 and 3.5-fold by all three RTKIs and PECAM-1 was down-regulated (*p* = 0.001) twofold by 5 µM ST (Fig. 3c). Corresponding changes were identified at protein expression level (Fig. 3d).

We next carried out directed differentiation of CPCs in vitro^18^, examining impacts on each differentiation pathway in the presence of each RTKI. This determined that ST caused a dose–response reduction in expression of α-sarcomeric actin, linked to cardiomyocyte lineage commitment, from 58.0 ± 1.5% in controls to 3.7 ± 1.2% after exposure to 5 µM ST (*p* < 0.001) (Fig. 3e,f). Furthermore, concentration-dependant reduction effects were seen with SM exposure for expression of smooth muscle actin from 63.1 ± 4.7% in controls to 35.8 ± 5.1% after exposure to 1.25 µM SM (*p* < 0.001) (Fig. 3g,h) and von Willebrand factor (linked to endothelial lineage commitment from 44.3 ± 6.9% in controls to 19.0 ± 3.8% after exposure to 1.25 µM SM (*p* < 0.001) (Fig. 3i,j). Therefore, RTKIs reduced the expression of molecules involved in the specification of CPCs, leading to a decrease in their differentiation potential into the endothelial, smooth muscle or cardiomyocyte lineage. Each RTKI was examined to determine impact on CPC differentiation into each lineage, although with no clear reduction in differentiation towards any lineage due to IM (Supplementary Fig. 5a–c). It was notable that IM in fact appeared to be correlated with an increase in smooth muscle differentiation (Supplementary Fig. 5b). For SM, there was no clear impact on differentiation to the cardiomyocyte lineage (Supplementary Fig. 5d), while ST caused reductions in smooth muscle cell differentiation comparable to SM (Supplementary Fig. 5e), but did not impact upon differentiation to the endothelial lineage (Supplementary Fig. 5f).

### RTKIs inhibit CPC expression of key regenerative growth factors and their second messengers

**Figure 4:**
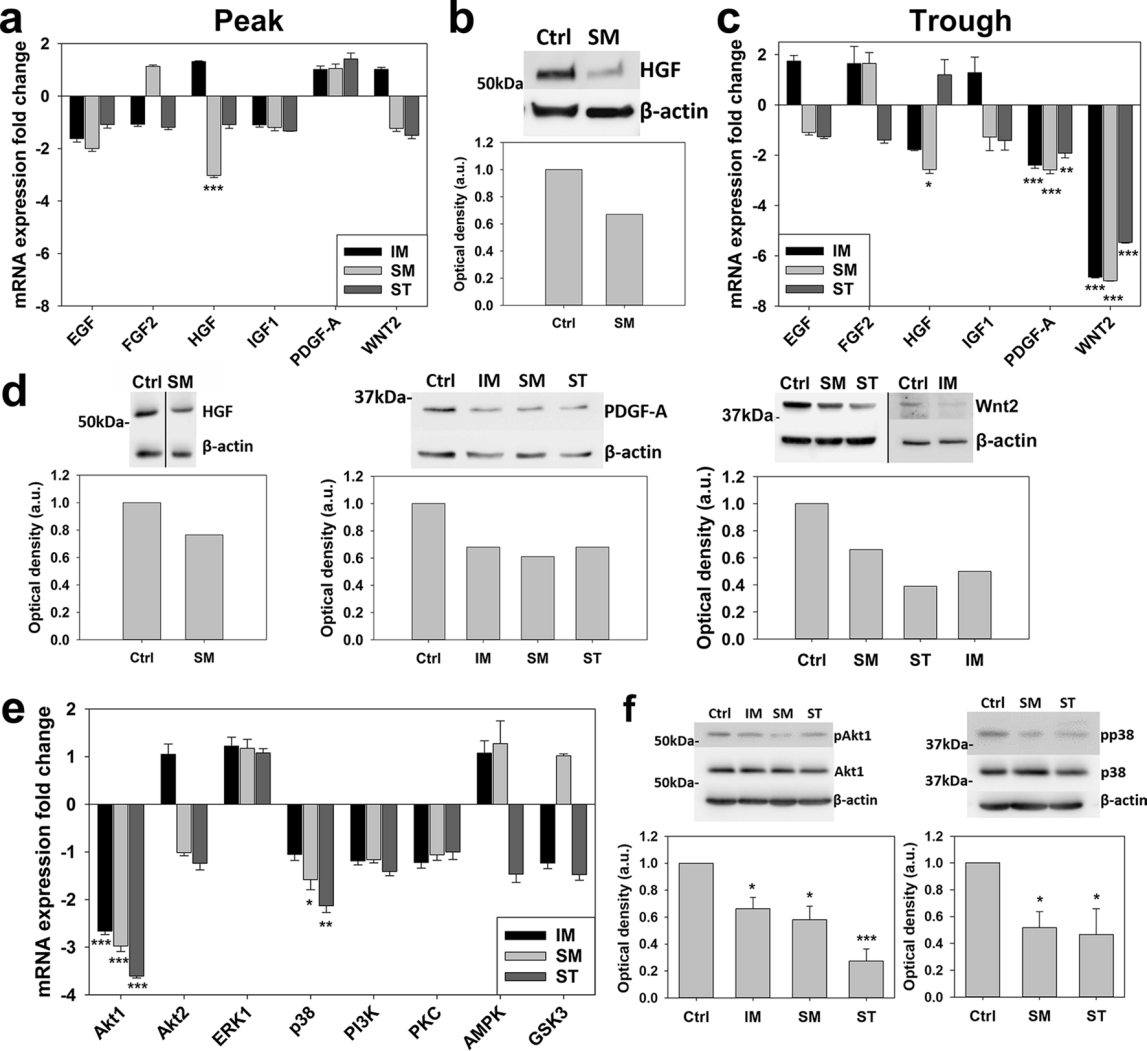
Pro-survival CPC secretome and second messengers inhibited by RTKIs. Real-time qPCR and Western blot analysis of RTKI effects on growth factor expression: (**a**) 10 µM SM for 24 h reduced HGF mRNA (n = 3), with (**b**) protein expression also assessed. HGF expression was reduced by IM or SM, PDGF-A and Wnt2 expression by 5 µM IM, 0.5 µM SM or 5 µM ST at 7 days, shown by (**c**) mRNA (n = 3) and (**d**) protein expression. Real-time qPCR and Western blot analysis of CPC second messenger expression following RTKI exposures: Akt1 expression levels reduced by 5 µM IM, 0.5 µM SM or 5 µM ST at 7 days, with p38 reduced by 0.5 µM SM or 5 µM ST at 7 days, with (**e**) mRNA (n = 3) and (**f**) level of phosphorylated protein/total protein normalized to the loading control (n = 3). All data are mean ± SEM; significance was determined by ANOVA with Holm-Šídák post hoc test to identify differences (*p < 0.05; **p < 0.01; ***p < 0.001). Blots cropped to relevant data, with optical density normalized to the loading control protein; full blot images presented in Supplementary Fig. 6. Panel (**d**) shows composite gels (for HGF and Wnt2), with all quantification using loading controls in the same gel and lane.

Due to the key role played by the CPC secretome in these cells’ reparative paracrine actions, we investigated the impact of RTKIs on selected regenerative growth factors (EGF, FGF2, HGF, IGF-1, PDGF-A and Wnt2)^17,21,29,43^. We found that exposure to 1.25 µM SM for 24 h (Fig. 4a) caused a threefold reduction (*p* < 0.001) in HGF mRNA expression, with HGF protein expression also assessed (Fig. 4b). At both mRNA and protein levels, exposure of CPCs to trough levels of RTKIs for 7 days caused greater decreases in growth factor expression (Fig. 4c,d), with PDGF-A, which was unaffected by peak levels (Fig. 4a) being reduced (*p* = 0.005) by ~ 2.5-fold (Fig. 4c,d). Moreover, WNT2 was also reduced (*p* < 0.001) by ~ sevenfold after exposure to all 3 RTKIs for 7 days (Fig. 4c,d).

We next characterised RTKI impacts on expression of linked second messenger systems. Exposure of CPCs to trough levels of all 3 RTKIs for 7 days caused significant (*p* = 0.002) reductions of > onefold in expression of the second messengers Akt1 and p38 signalling, which was translated to the protein level (Fig. 4e,f). Therefore, RTKI exposure reduces expression of pro-regenerative factors and their linked second messenger systems in CPCs.

### Specific second messenger blockade impacts on CPC characteristics

**Figure 5:**
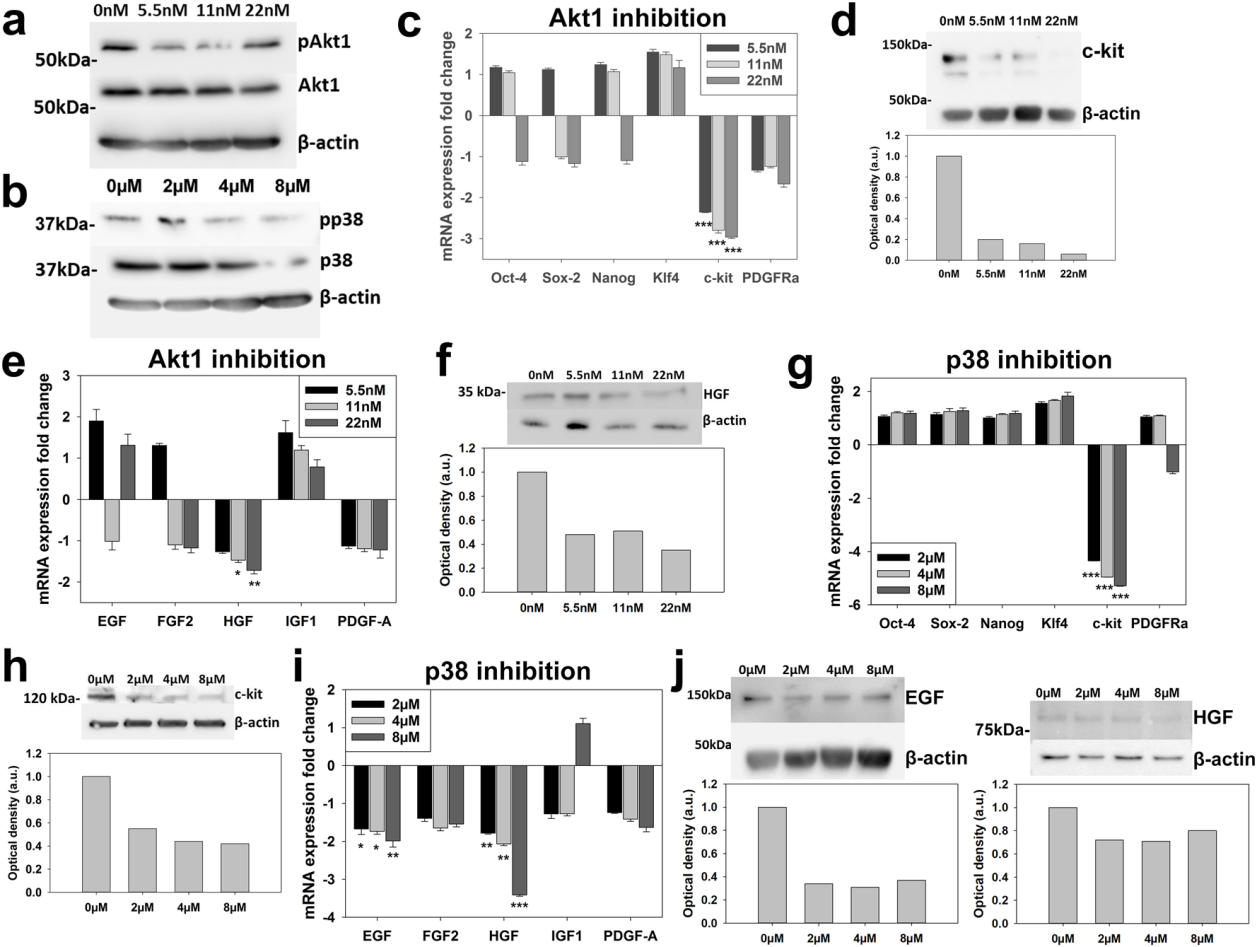
Specific second messenger blockade impacts on CPC characteristics. Application to CPCs of (**a**) Akt1 blocker A674563 or (**b**) p38 inhibitor SB203580 caused down-regulation of respective signalling pathways. (**c**) A674563 downregulated c-kit gene activity but did not impact on CPC ‘stemness’ genes or PDGFRα expression (n = 3), with (**d**) c-kit protein expression also assessed. (**e**) A674563 reduced expression of HGF mRNA (n = 3), with (**f**) protein expression also assessed. (**g**) SB203580 downregulated c-kit gene activity but did not impact on CPC ‘stemness’ genes or PDGFRα expression (n = 3), with (**h**) c-kit protein expression also assessed. (**i**) SB203580 significantly reduced EGF and HGF mRNA expression, with (**j**) protein expression also assessed. All data are mean ± SEM; significance was determined by ANOVA with Holm-Šídák post hoc test to identify differences (*p < 0.05; **p < 0.01; ***p < 0.001). Blots cropped to relevant data, with optical density normalized to the loading control protein; full blot images presented in Supplementary Fig. 6.

Our final objective was to identify any direct correlation between CPC characteristics and the second messenger systems inhibited by RTKIs, by carrying out specific blockade of the p38 and Akt1-signalling. The specific Akt1 blocker A674563 and specific p38 inhibitor SB203580 were each applied over a range of doses to CPCs over 7 days. We first confirmed that application of these inhibitors reduced signalling through the Akt1 (Fig. 5a) and p38 (Fig. 5b) pathways.

Akt1 inhibition did not significantly alter mRNA expression of stemness pluripotency-associated genes (Fig. 5c), but reduced c-kit (*p* < 0.001) expression two to threefold at all doses applied (Fig. 5c,d). Akt1 blockade also reduced (*p* < 0.05) expression of HGF at mRNA and protein levels (Fig. 5e,f).

Examination of p38 inhibition showed comparable effects, with no impact seen on pluripotency-associated genes or PDGFRα (Fig. 5g), but c-kit mRNA expression reduced (*p* < 0.001) four to fivefold at all doses applied (Fig. 5g), which was translated to a reduction at the protein level (Fig. 5h). Further investigation showed that p38 inhibition reduced (*p* < 0.05) growth factor mRNA expression (Fig. 5i) and Western blotting verified reduced EGF and HGF protein expression with p38 inhibition (Fig. 5j). Therefore, blocking second messenger systems inhibited by RTKIs in CPCs, reduced c-kit, and the growth factors EGF and HGF.

## Discussion

The findings of this study confirm our hypothesis that commonly-used RTKIs with known cardiotoxicity impact significantly on CPC survival, and also negatively affect the pro-reparative characteristics of these cells. Only a few previous studies have examined RTKI effects on CPCs, with these predominantly focused solely on imatinib^37^ or trastuzumab^44^, while this study demonstrates effects on CPCs from a range of RTKIs. Interestingly however, the study of trastuzumab (in cardiosphere-derived cells) identified reduced angiogenic potential in these cells when applied in vitro, coupled with reduced angiogenesis seen in post-MI myocardium following drug application in vivo^44^.

The reduction of CPC numbers seen here due to reduced cell viability (Fig. 1) and lowered proliferation (Fig. 2) is consistent with findings in rat CPCs following IM treatment in vivo and in vitro^45^. However, that same study found a contrasting result for human CPCs, with only moderately decreased growth over 6–24 h, and none seen at 48 h^45^. While CPC isolation methods appear similar to those used here, that study also carried out a parallel experiment in human leukaemia cells expressing high levels of c-kit, identifying a greater IM effect, which suggests the reduced sensitivity to IM seen in their human CPC population could be related to a lower level of c-kit expression. It has been shown in a human leukaemia cell line that IM causes c-kit to internalise and undergo lysosomal degradation^46^. Investigation of the c-kit ligand (stem cell factor) role in CPCs found potentiation of proliferation and migration^47^, entirely consistent with our findings when the RTK, c-kit, is blocked by IM, SM or ST (Fig. 2). Although the roles of VEGFRs and/or PDGFRs on CPC proliferation were not investigated in that study, the authors did note a positive impact by VEGF on CPC migration, suggesting a valuable role for these RTKs in the CPC injury response. An interesting comparison is with myocardial IGF-1 overexpression reducing CPC senescence, which otherwise occurs with age and other common cardiovascular diseases, including diabetes^48^. While we saw no significant RTKI impact on IGF-1 expression in CPCs here, the reduced CPC cycling seen could precede senescence. Longer-term study of RTKIs impacts on CPCs would be of value in clarifying this.

While the doses used here reflect those identified in clinical plasma levels, a more accurate approach would be to apply those doses found in the interstitium, which are all-but-certainly notably lower than those found in plasma, ideally in a varying wave-form pattern reflecting the changes seen with the administered dose peaking and descending over a few hours. This would require a far more extensive study than this, focused specifically on identifying the potential for RTKIs to impair CPC function, but would be essential for advancing this work towards clinical benefit.

Our use of human CPCs was to ensure that the in vitro analyses, the main study focus, were of maximum translational relevance. This precluded analysis of RTKI toxicity to these cells in their normal environment in situ; hence we carried out a defined parallel study of the most CPC-toxic RTKI, to confirm this CPC toxicity was replicated in vivo, using a clearly cardiotoxic dose. This was shown, with CPC numbers reduced as seen with doxorubicin toxicity^35^, although subsequent analysis of our cardiac functional data revealed a possible correlation between CPC numbers in situ and cardiac functional recovery (Fig. 1). The in vivo protocol duration excluded CPC differentiation as a factor, but loss of CPC support for injured cells or tissue is a potential link. Confirmation or exclusion of this was beyond this study’s scope but could be a useful course of investigation, to explain this observation and clarify CPC role in cardiac recovery from diffuse injury. It is also possible that the non-recovery population of rats suffered a more severe direct action of RTKI toxicity on the cardiomyocytes, which is the principal route by which cardiac function reduction takes place. This could have induced an injury too severe for endogenous repair mechanisms to cope with, whether coupled with a reduced CPC population or not.

While this model effectively demonstrated that RTKI-induced CPC toxicity was reproduced in vivo, it was limited in that it does not recapitulate the RTKI-induced cardiotoxicity seen clinically, being primarily focused on affirming CPC toxicity. In addition, the administration of the RTKI via intraperitoneal injection contrasts with the oral administration seen in human patients, with likely according variances in drug plasma levels.

Evidence to date mostly supports the main function of c-kit as being in maintenance of cell proliferation, while the role of VEGFRs in CPCs is less clearly-defined. Much work has been done on the role of VEGFRs in driving angiogenesis^49^, suggesting a plausible role for these receptors in CPC vascular cell lineage generation. The findings here strongly support this in the case of SM at least, which blocks all three VEGFRs and caused significant dose-related reductions in the CPC ability to form endothelial or smooth muscle cells (Fig. 3). It was also notable in this regard that IM, which does not target VEGFRs, was associated with increased CPC smooth muscle cell differentiation (Supplementary Fig. 5). These data are notable in light of the roles of SM and ST in repressing VEGFR stimulation and particularly that VEGF signalling repression can cause a reversible cardiomyopathy due to loss of capillary density (for review, see Bair et al.^50^). This would be exacerbated in the case of SM and ST toxicity by their simultaneous repression of PDGFR-driven pro-angiogenic signalling from cardiomyocytes^51^.

When considering the mechanisms underlying the broad impact of RTKIs on CPC differentiation, with reduced generation of all three lineages studied (Fig. 3), it is notable that all three RTKIs also reduced expression of Wnt2 (Fig. 4). In embryonic stem cells, Wnt2 drives cardiomyogenic differentiation^52^, and is required for the formation of smooth muscle cells in developing lung mesenchymal tissue^53^. Further, the Wnt-β-catenin pathway is upregulated in the early phase of satellite cell myogenic differentiation^54^. This points to a critical role for Wnt2 in early stem/progenitor cell commitment, consistent with the loss of CPC auto- or paracrine Wnt2 signalling reducing CPC differentiation across multiple lineages.

We have previously determined that HGF increases pig CPC migration, proliferation, and myogenic specification in vitro^17^. Moreover, intra-coronary IGF-1 and HGF application in the pig significantly improves both cardiomyocyte survival and cardiac function after ischaemic injury^17^. This is reinforced by findings that HGF over-expression in mesenchymal stem cells (MSCs) potentiates their regenerative capabilities when applied to ischaemic myocardium, with improved function, increased angiogenesis and reduced scar size^55^. Considering the impact of IM and SM on HGF expression seen here (Fig. 4), it is notable that HGF can drive CPC migration^17,56^ and it was identified that IM reduces CPC migration in vitro^57^. Our findings indicate that the mechanism impacting on CPC migration could be prolonged IM exposure reducing HGF auto- and paracrine signalling. A notable limitation is that we did not have scope within this study to characterise their mechanisms of secretion, for example to identify whether the factors are secreted freely as molecules into the medium or tissue interstitium, or are released within exosomes/microvesicles. These are questions of great interest and translational relevance, and warrant further investigation.

Examination of RTKI impact on linked second messenger systems identified significantly reduced Akt1 expression (Fig. 4), and selective blockade of which caused reduced HGF expression (Fig. 5). With strong interest in the reparative potential of CPC secretome, it is notable that a recent study^58^ found Akt over-expression in MSCs produced exosomes with significantly higher pro-regenerative potential. This indicates scope to use RTK signals to generate maximally regenerative CPC secretome, such as a secretome rich in pro-survival growth factors and cytokines, building on previous work^17,30,59^, to prevent cardiomyocyte death post-injury. An additional, complementary route for exploitation would be a maximally angiogenic CPC secretome, possibly via targeted VEGFR stimulation^60^.

Another finding was that SM and ST each reduced p38 signalling in CPCs (Fig. 4), with p38 blockade reducing CPC growth factor expression (Fig. 5); the p38 pathway was identified as key for pro-angiogenic VEGF signalling released by cardiomyocytes under hypoxic stress, both in vitro and in vivo^61^. Although no impact was seen on VEGF expression here, that study emphasises the importance of cell–cell signalling between different cells of the myocardium, one of the fundamental views underlying this present work. An important question, which this study was not able to investigate fully, was whether there is a correlation between individual RTK activity and specific CPC functions. While the effects of the RTKIs examined here on their target RTKs’ activity could point towards this, a fuller answer could be given by examining correlations between an array of many RTKIs and impacted cellular functions: this is one path for our intended further study.

Another potential route by which RTKIs may impact upon CPC biology is inhibition of fms-like tyrosine kinase 3, or Flt3, which is known to be inhibited by both SM and ST^62^. This receptor has been shown very recently to play a role in mouse side population CPCs, with knock-out of this gene reducing side population CPC numbers and their ability to generate endothelial cells in vitro and myocardial capillaries in vivo^63^. Although Flt3 expression was not shown in the CPCs examined here, the side population findings identify it as a target of interest.

While the main focus of examination of CPCs has been on their role as a positive contributor to myocardial recovery from disease, a notable recent finding was their role in the genesis of atrial myxomas^64^. The similarities between these myxoma CPCs and those of normal myocardium raises the issue of whether CPC loss may be an unavoidable penalty of successful anti-cancer therapy. This could be addressed by investigating whether the differences between myxoma and ‘normal’ CPCs^61^ alter their respective vulnerabilities to RTKIs.

To determine a CPC-protective co-therapy, precisely this kind of comparative study would be necessary, to preclude that an effectively CPC-protective co-treatment did not also protect tumour cells. While the aforementioned myxoma-generating cells would be one such comparison, the other would plainly be the target tumour cell type. Therefore, the recent finding that 3-hydroxy-3-methylglutaryl coenzyme A reductase inhibitors (‘statins’) can drive CPC proliferation while reducing apoptosis^65^ is of interest, especially as the apoptotic stimulus blocked was Akt inhibition. These drugs would be good candidates for such a comparative study, as this extensively-used drug class is already well-characterised regarding patient safety profile.

In summary, this study demonstrated a range of impacts caused by clinically-used cardiotoxic RTKIs on human CPCs, reducing their supportive and reparative capacities. We further identified that reduced ability to recover from RTKI cardiotoxicity in vivo was correlated with reduced numbers of CPCs in situ.

## Supplementary Information

Supplementary Information.
