# Supplementary material for: Receptor tyrosine kinase inhibitors negatively impact on pro-reparative characteristics of human cardiac progenitor cells
Source: Sci Rep. 2022 Jun 16;12:10132. doi: 10.1038/s41598-022-13203-3 (PMC9203790; doi:10.1038/s41598-022-13203-3)
Supplement: Supplementary file 1 — Supplementary Information. [file 41598_2022_13203_MOESM1_ESM.pdf]

Receptor tyrosine kinase inhibitors negatively impact on pro-reparative characteristics of human cardiac progenitor cells

Andrew J. Smith, Prashant Ruchaya, Robert Walmsley, Kathleen E. Wright, Fiona C. Lewis-McDougall, Jacquelyn Bond, Georgina M. Ellison-Hughes

SUPPLEMENTARY FIGURE 1

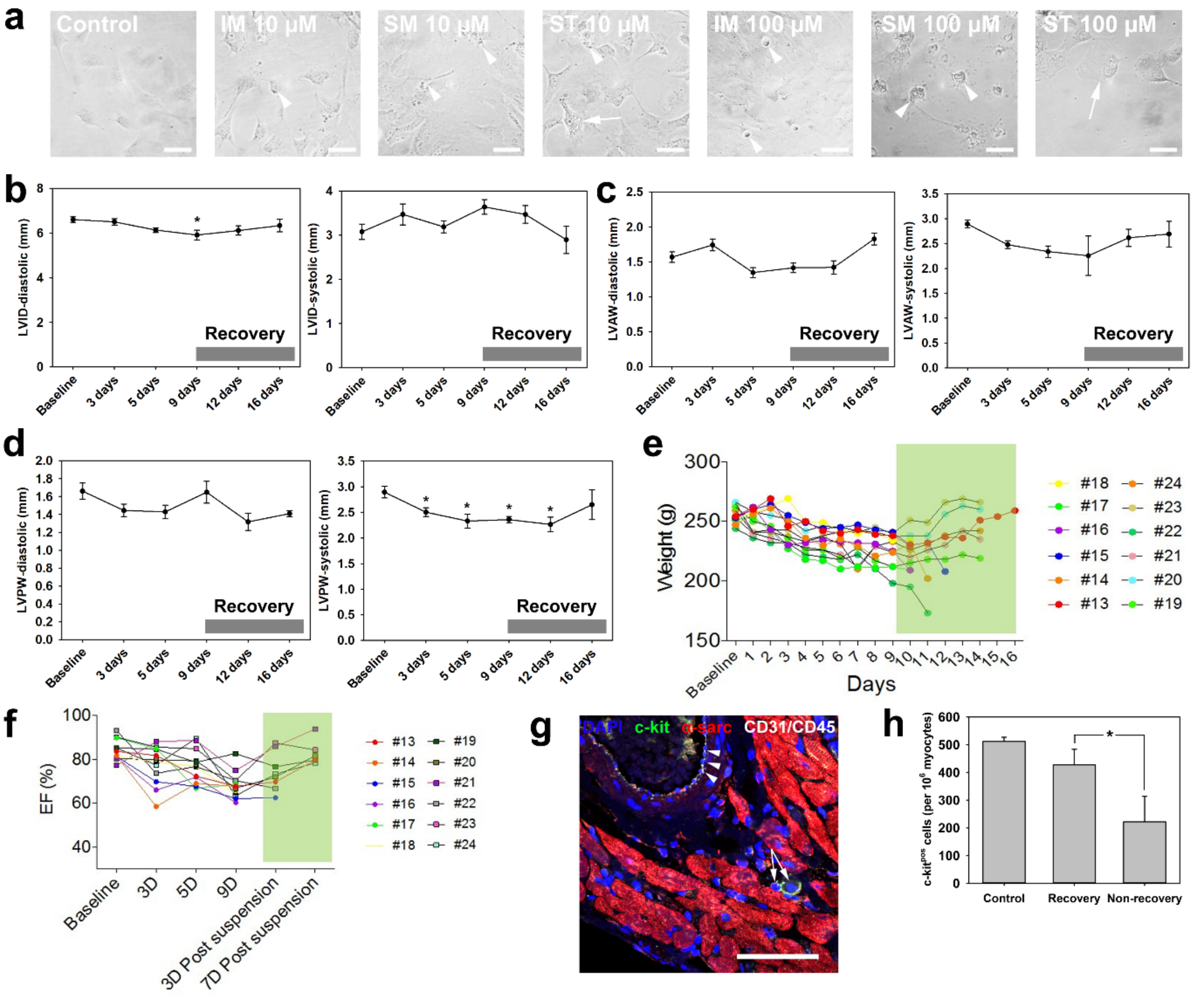

Supplementary Figure 1 - RTKIs impact upon CPCs *in vitro* and *in vivo*. Morphological appearances of *in vitro* human CPCs treated with RTKIs were consistent with viability findings **(a)**, with dead cells (arrowheads) and severely-damaged cells (arrows); scale bar=10 µm. Application of SM to adult male Wistar rats *in vivo* caused **(b)** transient reduction in left ventricular internal diameter during diastole, **(c)** no change to left ventricular anterior wall thickness and **(d)** reduced left ventricular posterior wall thickness transient that resolved after cessation of SM (n=12). In addition, SM caused **(e)** weight reduction over the course of 9 days' treatment followed by recovery for 7 days (individual data series, total n=12). Echocardiography analysis **(f)** produced a comparable set of individual data series for cardiac ejection fraction (individual data series, total n=12). Green areas indicate recovery period after RTKI application. **(g)** Representative immunostaining image: α-sarcomeric actin (red), c-kit (green), DAPI (blue) CD31 and CD45 (white) showing CPC *in situ* (arrow) negative for both CD31 and CD45, with CD31/CD45-

positive cells in nearby vascular tissue (arrowheads); scale bar=50 μm. **(h)** Analysis of CPC numbers per 10^6 cardiomyocytes showed a significant reduction in non-recovery animals (p<0.05, n=6).

SUPPLEMENTARY FIGURE 2

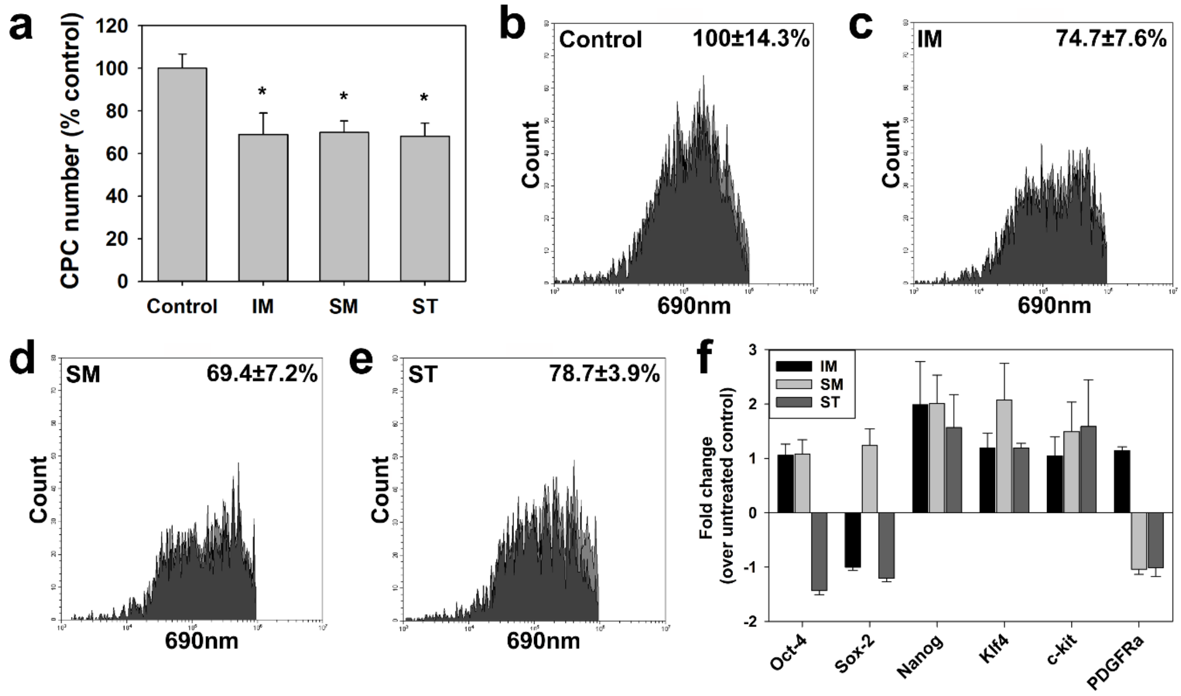

Supplementary Figure 2 - RTKIs reduce CPC cell numbers but not 'stemness'. Analysis of viable CPC numbers after 48 hours RTKI treatment, with **(a)** dead cells excluded by Trypan blue staining (n=4). Further analysis of viable CPC numbers after 48 hours RTKI treatment, with **(b-e)** dead cells excluded by propidium iodide staining (detected by 690 nm emission): representative flow cytometry plots of control and RTKI-treated CPCs (percentages of untreated control CPC viable cell counts in corner, n=3). **(f)** Real-time qPCR analysis of CPC 'stemness' and CPC marker gene expression after 24 hours (n=3). All graph data are mean±SEM.

SUPPLEMENTARY FIGURE 3

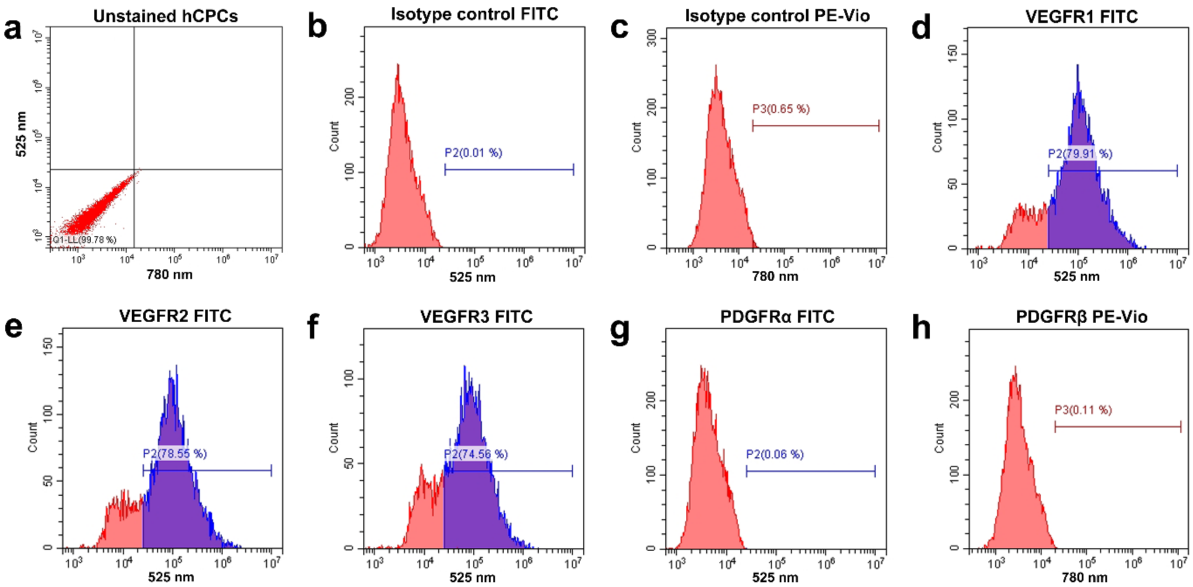

Supplementary Figure 3 - CPC population expression of RTKs. Live, unfixed CPCs were analysed by flow cytometry to determine expression of VEGFRs and PDGFRs. **(a)** Background fluorescence in both channels (525 and 780 nm) were minimal, also when CPCs were stained by isotype control antibodies conjugated with **(b)** FITC or **(c)** PE-Vio. Expression levels in CPCs of **(d)** VEGFR1, **(e)** VEGFR2, **(f)** VEGFR3, **(g)** PDGFRα and **(h)** PDGFRβ.

SUPPLEMENTARY FIGURE 4

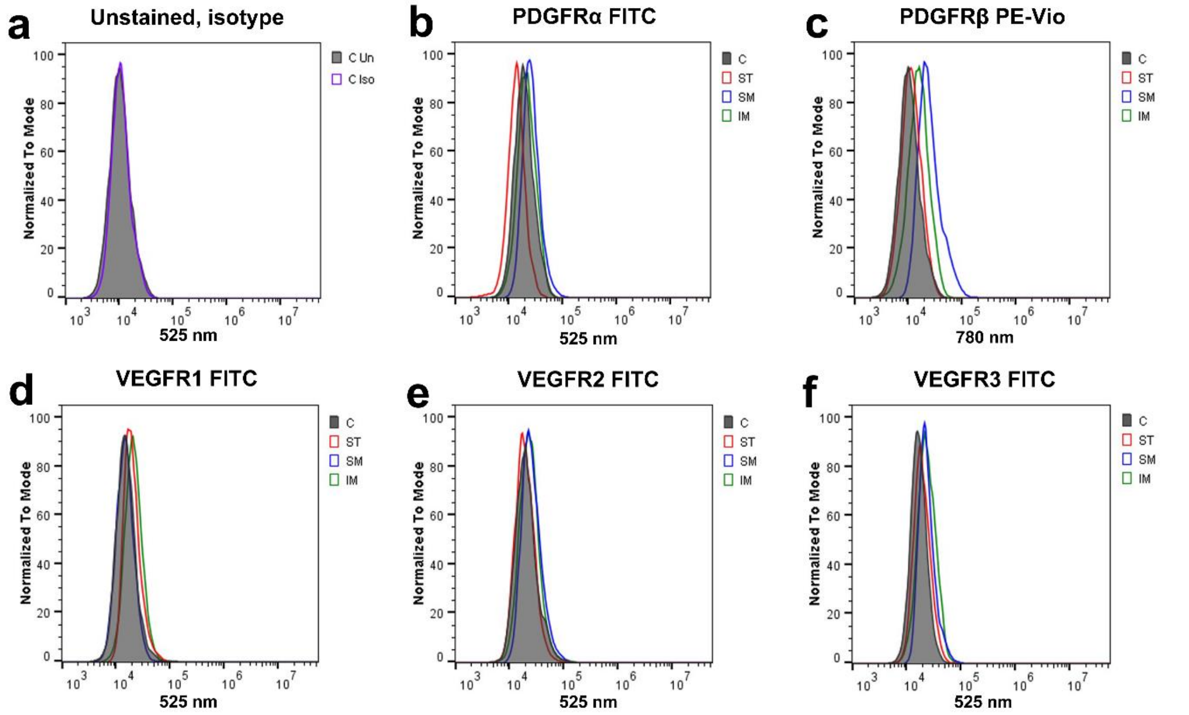

Supplementary Figure 4 - CPC population expression of RTKs following RTKI exposure. Live, unfixed CPCs were analysed by flow cytometry to determine expression of VEGFRs and PDGFRs, following treatment with 5 µM IM, 0.5 µM SM or 5 µM ST. Expression levels in treated and untreated CPCs of **(a)** unstained CPCs, **(b)** PDGFRα, **(c)** PDGFRβ, **(d)** VEGFR1, **(e)** VEGFR2 and **(f)** VEGFR3.

SUPPLEMENTARY FIGURE 5

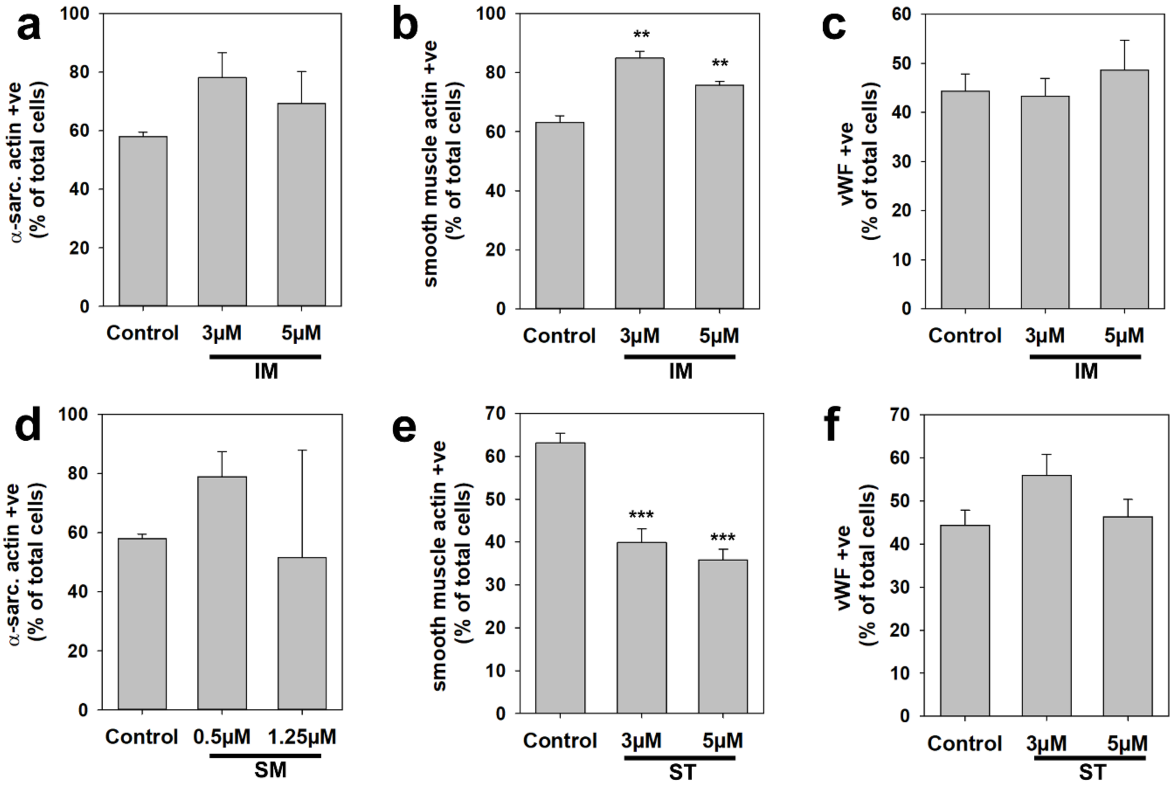

Supplementary Figure 5 - CPC differentiation potential following RTKI exposure. Directed differentiation of CPCs *in vitro* determined that: **(a)** percentage of CPCs expressing α-sarcomeric actin was unaffected by IM (n=4); **(b)** percentage of CPCs expressing smooth muscle actin increased in the presence of IM (n=4); **(c)** percentage of CPCs expressing von Willebrand factor was unaffected by IM (n=4); **(d)** percentage of CPCs expressing α-sarcomeric actin was unaffected by SM (n=4); **(e)** percentage of CPCs expressing smooth muscle actin was reduced by ST (n=4); **(f)** percentage of CPCs expressing von Willebrand factor was unaffected by ST (n=4). All data are mean±SEM; significance was determined by ANOVA with Holm-Šídák *post hoc* test to identify differences (***p*<0.01; ****p*<0.001).

SUPPLEMENTARY FIGURE 6 - Western blot raw image files

Figure 2g (c-kit)

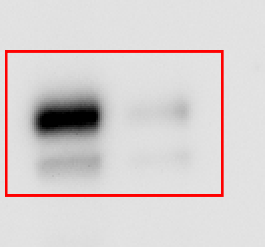

Figure 3b (Nkx2.5)

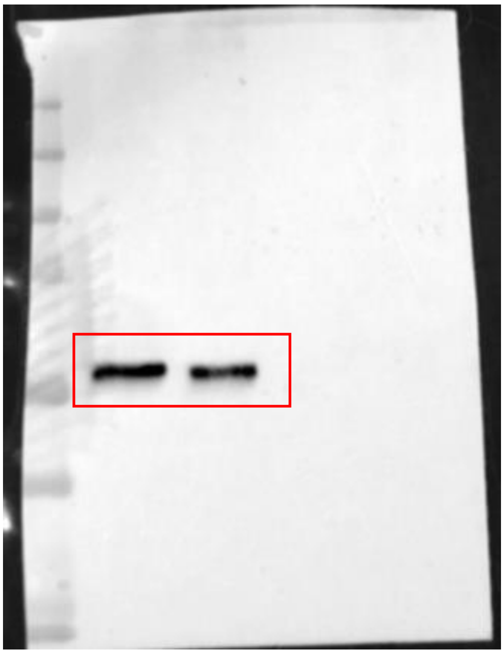

Figure 3d (PECAM-1)

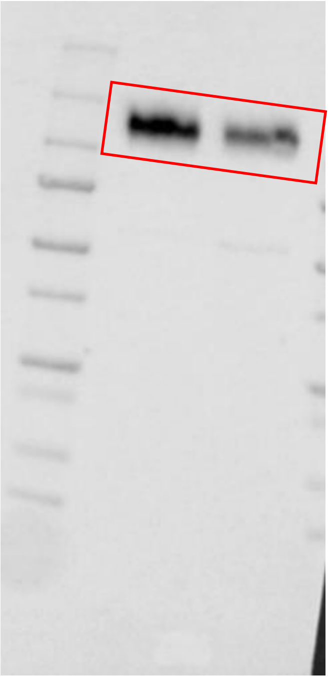

Figure 3d (Nkx2.5)

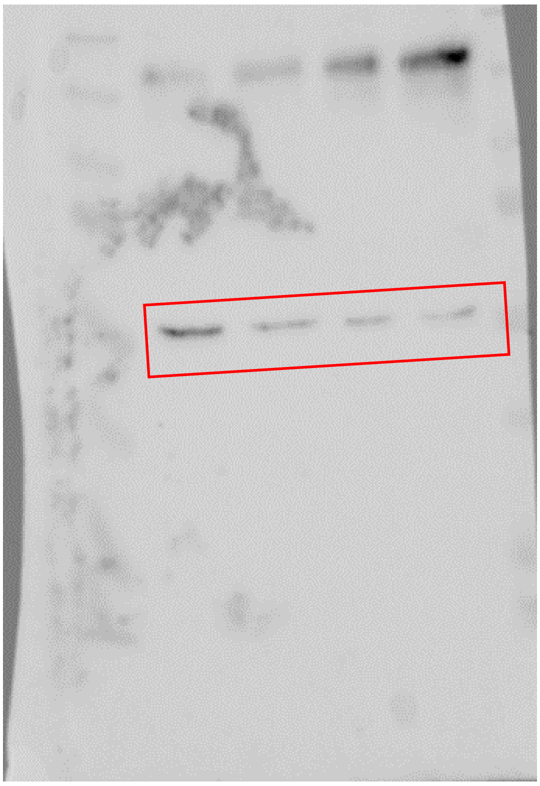

Figure 4b (HGF)

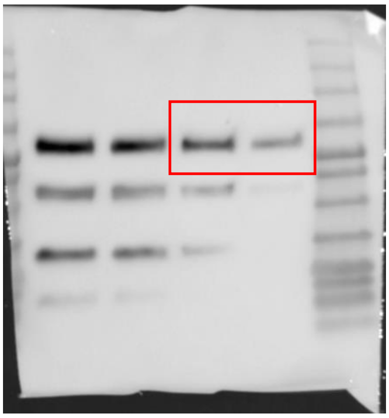

Figure 4d (HGF)

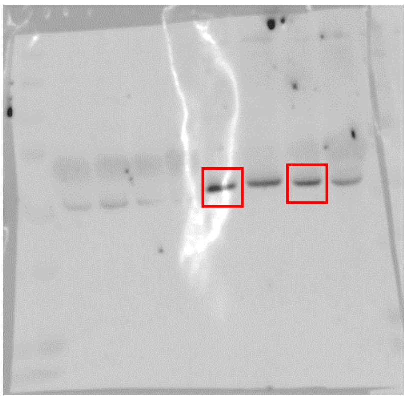

Figure 4d (PDGF-A)

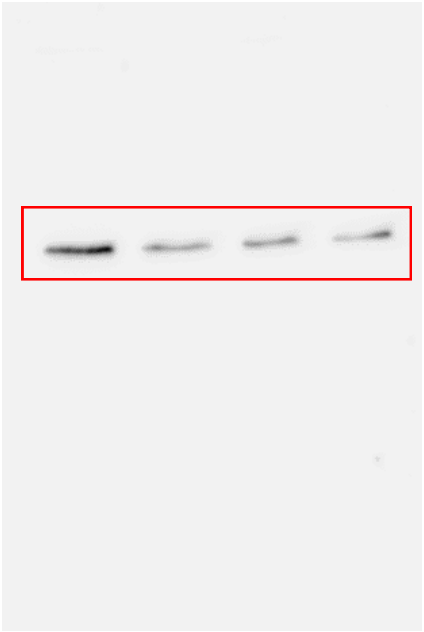

Figure 4d (Wnt2)

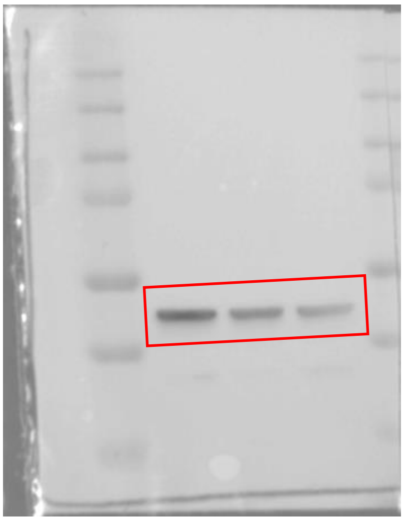

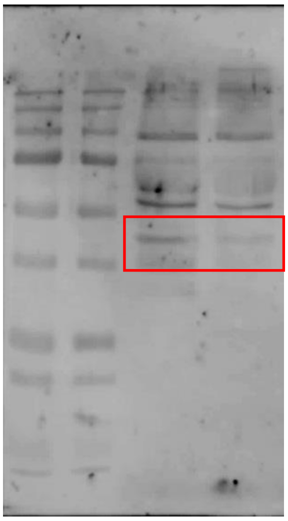

Figure 4f (Akt1, pAkt1)

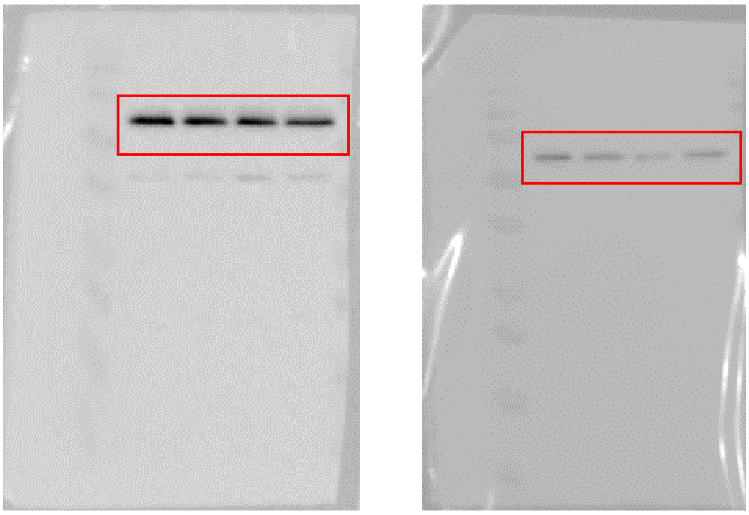

Figure 4f (p38, pp38)

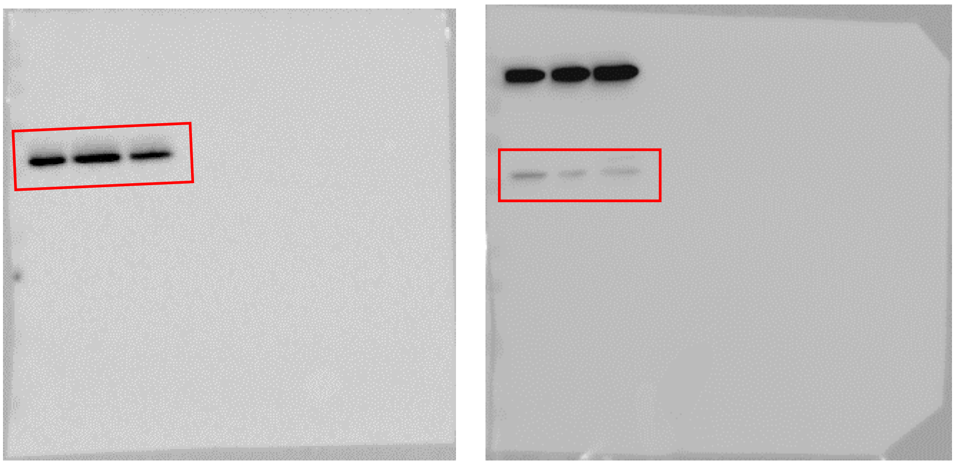

Figure 5a (Akt1, pAkt1)

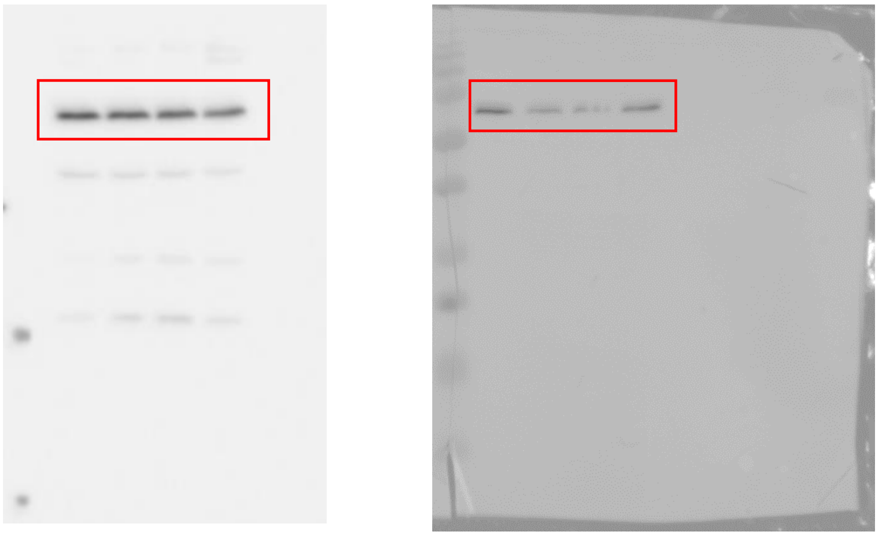

Figure 5b (p38, pp38)

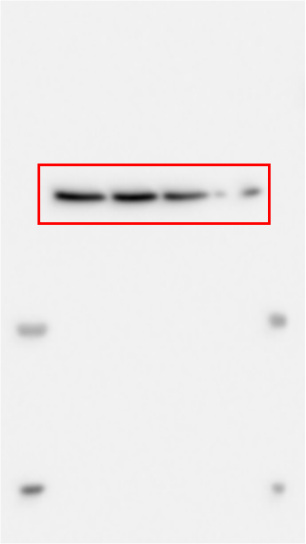

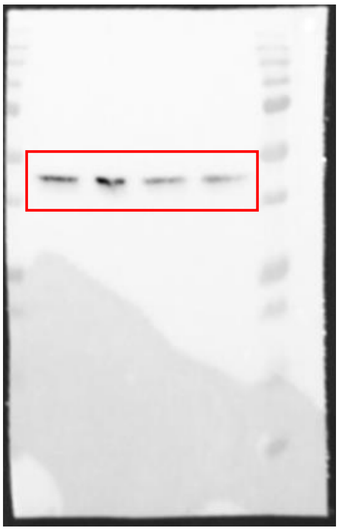

Figure 5d (c-kit)

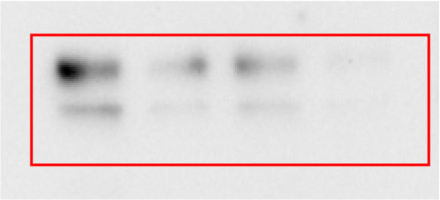

Figure 5f (HGF)

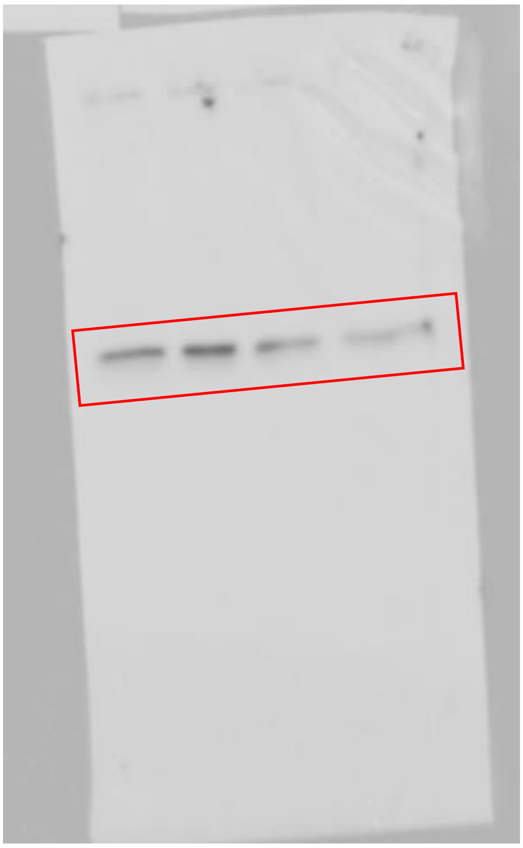

Figure 5h (c-kit)

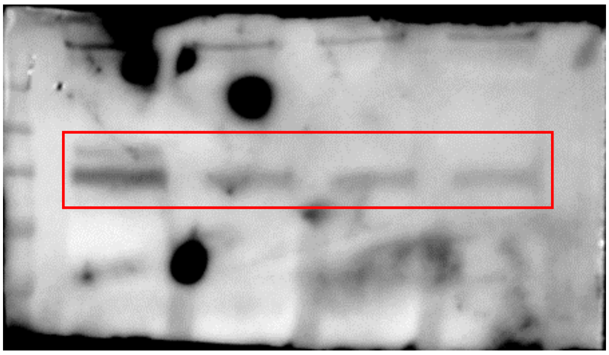

Figure 5j (EGF)

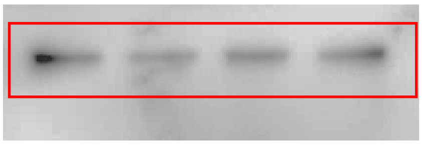

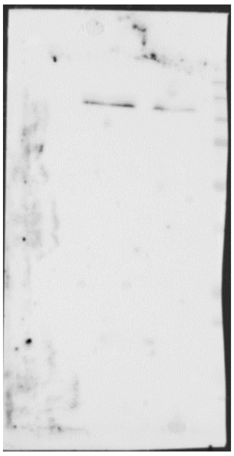

Figure 5j (HGF)

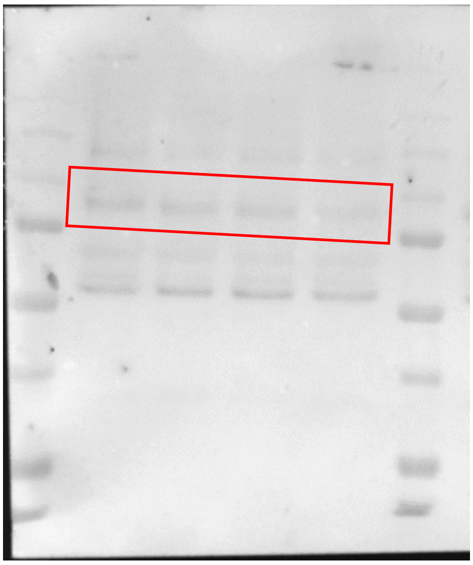

SUPPLEMENTARY FIGURE 6 - Western blot β-actin raw image files

Figure 2g (c-kit)

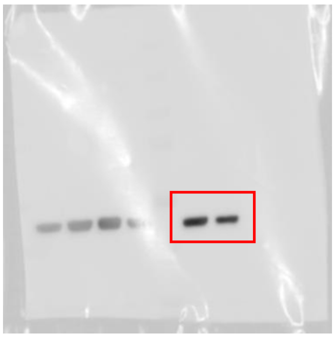

Figure 3b (Nkx2.5)

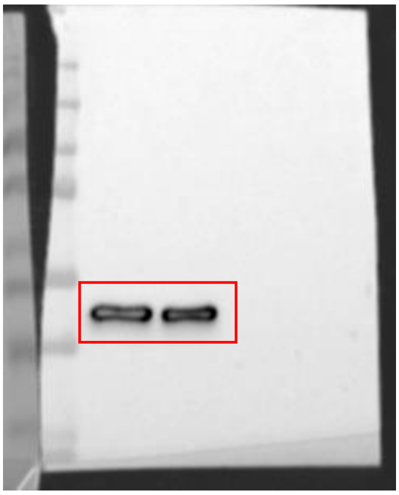

Figure 3d (PECAM-1)

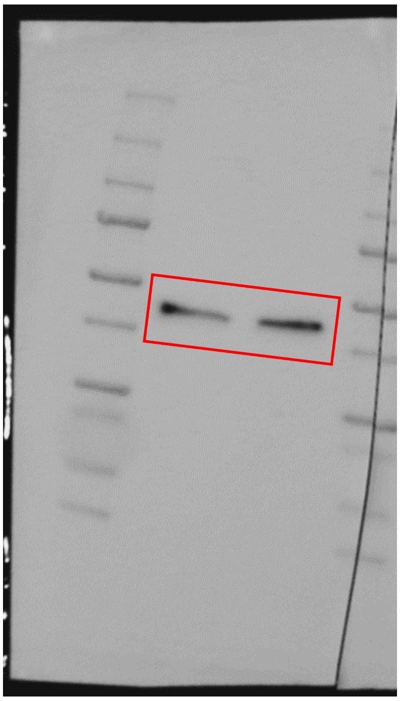

Figure 3d (Nkx2.5)

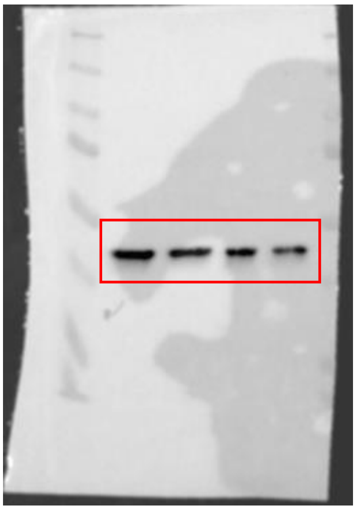

Figure 4b (HGF)

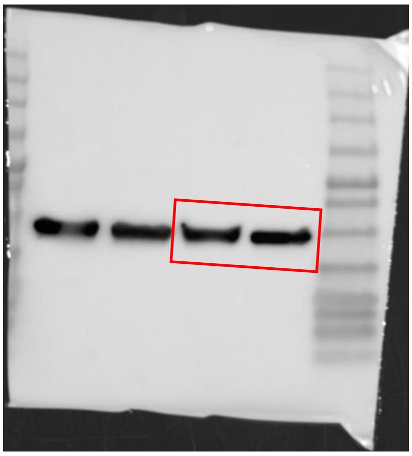

Figure 4d (HGF)

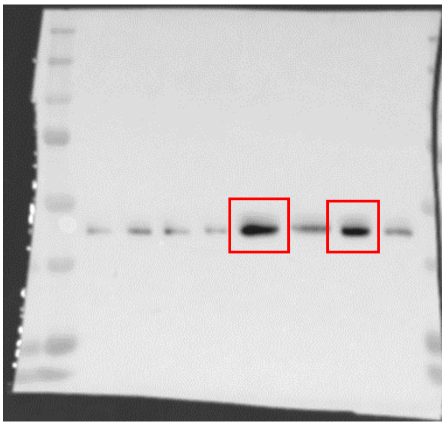

Figure 4d (PDGF-A)

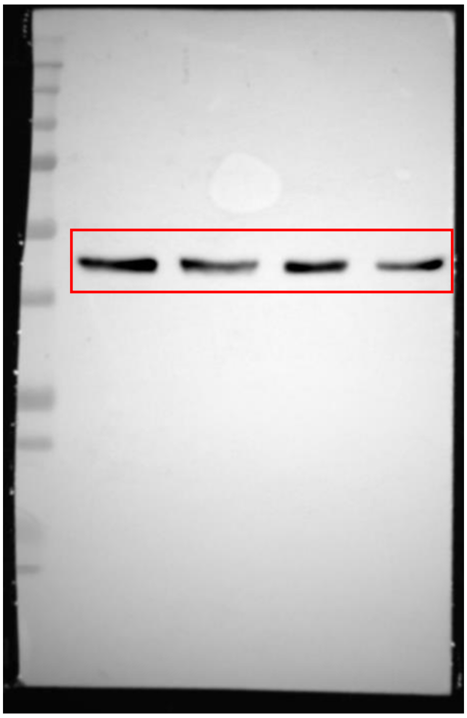

Figure 4d (Wnt2)

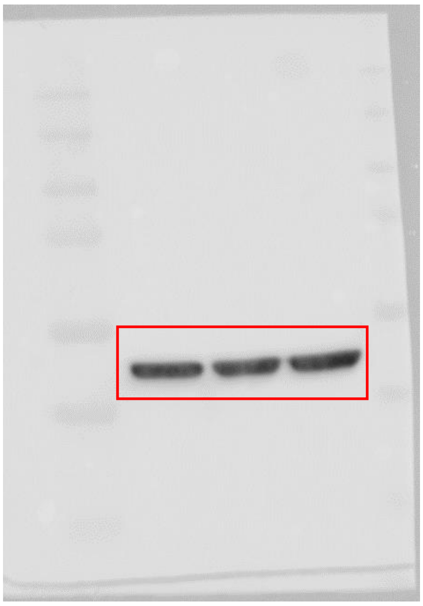

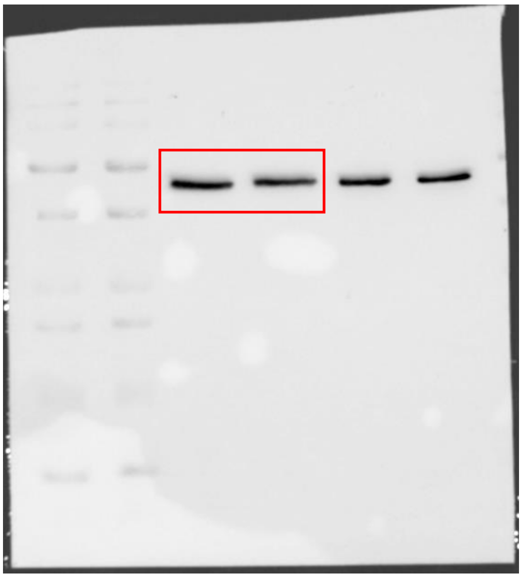

Figure 4f (Akt1)

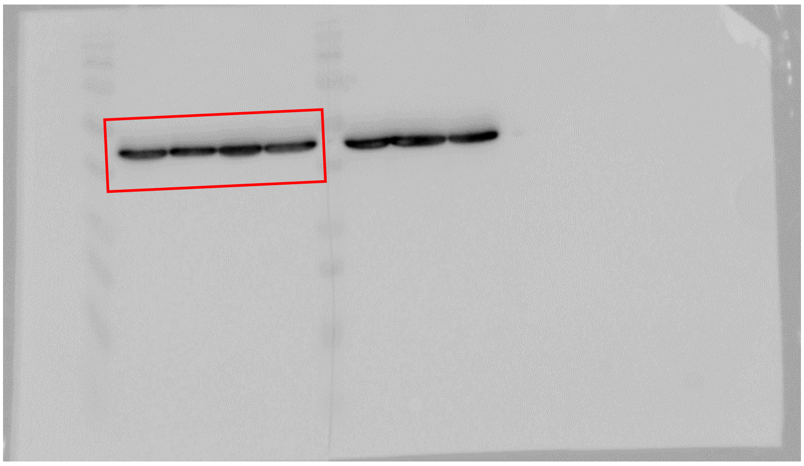

Figure 4f (p38)

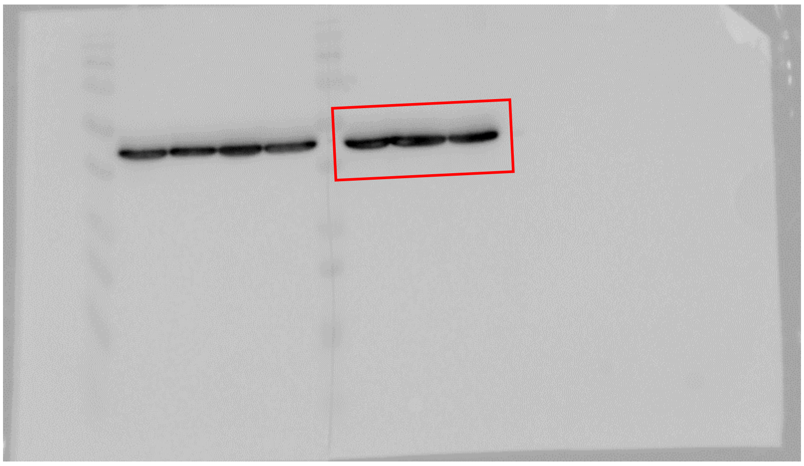

Figure 5a (Akt1)

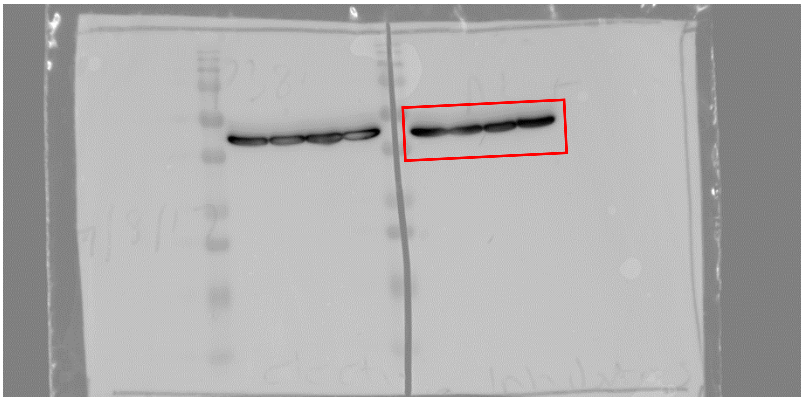

Figure 5b (p38)

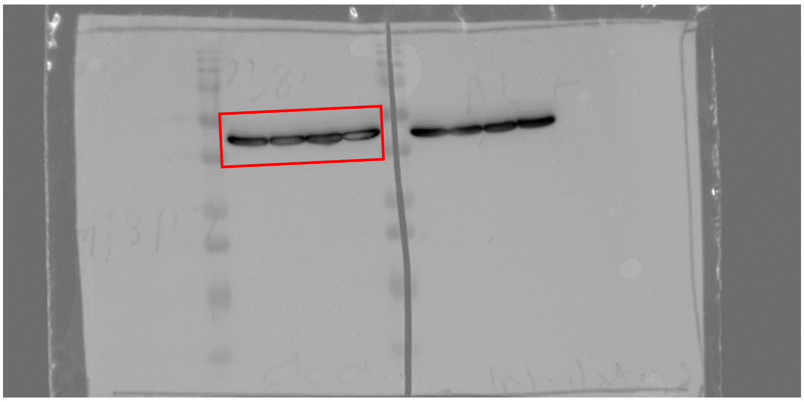

Figure 5d (c-kit)

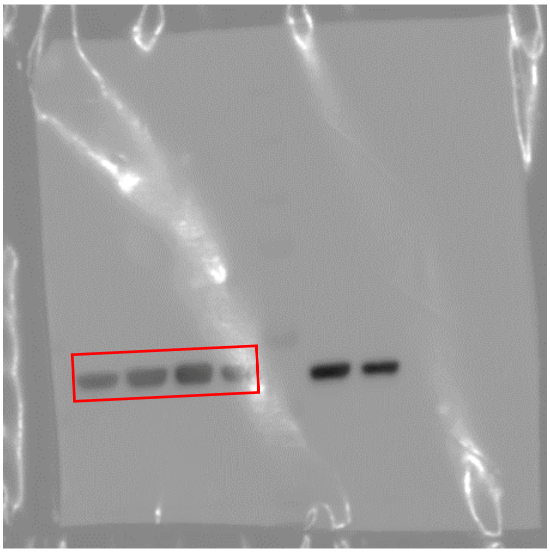

Figure 5f (HGF)

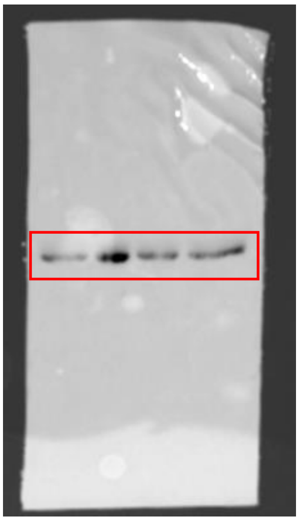

Figure 5h (c-kit)

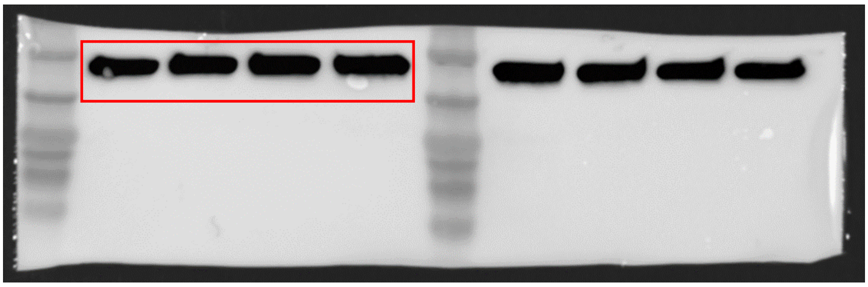

Figure 5j (EGF)

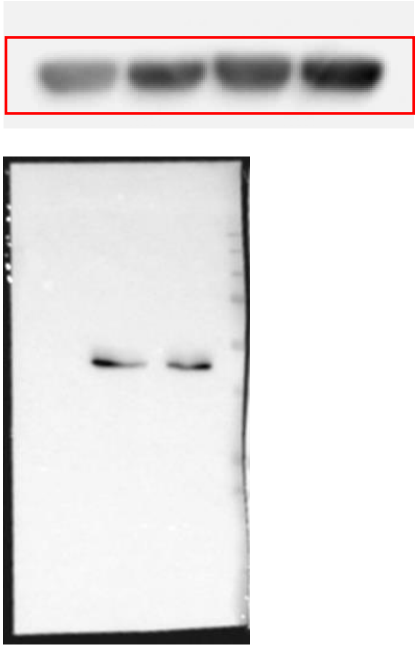

Figure 5j (HGF)

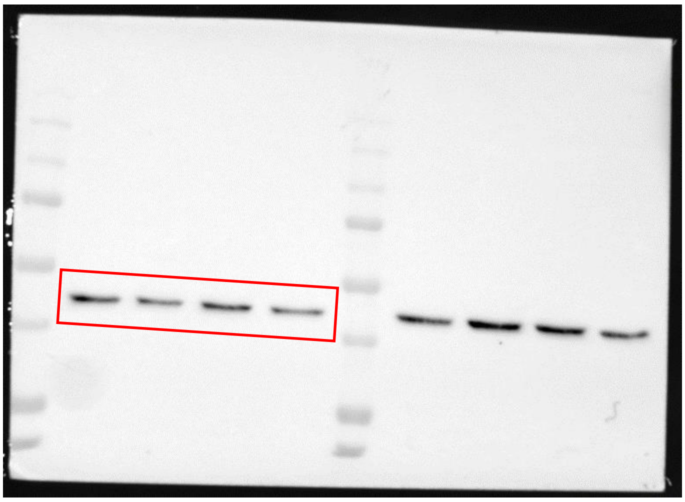

Supplementary Table 1 - primer sequences

Target gene	Forward sequence (5'-3')	Reverse sequence (3'-5')	Accession number
Oct-4	GATGGCGTACTGTGGGCCC	CAAAACCCGGAGGAGTCCCA	NM_002701.6
Sox-2	GGGGAAAGTAGTTTGCTGCC	CGCCGCCGATGATTGTTATT	NM_003106.4
Nanog	TGGTGTGACGCAGAAGGC	TGCACCAGGTCTGAGTGTTC	NM_024865.4
Klf4	AAGCCAAAGAGGGGAAGACG	CATGTGTAAGGCGAGGTGGT	NM_001314052.2
c-kit	AGGAAACGCTCGACTACCTG	CATTCCAGGATAGGGGCTGC	NM_001385286.1
PDGFRα	CGGAGGAGAAGTTTCCCAGAG	CTGCTCACTTCCAAGACCGT	NM_001347827.2
GATA-4	CGACACCCCAATCTCGATATGTT	ACAGATAGTGACCCGTCCCA	NM_002052.5
GATA-6	AAGCGCGTGCCTTCATCA	CATAGCAAGTGGTCTGGGCA	NM_005257.6
PECAM-1	GAAATGTCCAGGCCAGCAGT	ATCTGCTTTCCACGGCATCA	NM_000442.5
Nkx2.5	CAAGTGTGCGTCTGCCTTTC	CACAGCTCTTTCTTTTCGGCTC	NM_004387.4
EGF	GGGAGCCTGAGCAGAAACTT	CTACAGGGCACGTGCAGTAA	NM_001963.6
FGF-2	CAAGCGGCTGTACTGCAAAA	TAGCTTGATGTGAGGGTCGC	NM_001361665.2
HGF	CAATGCCTCTGGTTCCCCTT	GGCAAAAAGCTGTGTTCGTG	NM_000601.6
IGF-1	CTCCCCTGTCTTCCCAGAGA	GTGCCCAACTACAACATCCCT	NM_000875.5
PDGF-A	TGTTTCTCCTCCTCCTGGCT	GGATCTCGGCTTCCTCGG	NM_002607.6
WNT2	CTCCGAAGTAGTCGGGAATCTG	TGCCAGCTCTGTTGTTGTGA	NM_003391.3
Akt1	GCATCCTGGTCCTGTCTTCC	CTGGCCACAGCCTCTGATG	NM_005163.2
Akt2	AAGAAGGCTGGCTCCACAAG	CATCCACTCCTCCCTCTCGT	NM_001626.6
ERK1	GTCAGACTCCAAAGCCCTTGA	TGTCTGTCTGGGCTAGGGG	NM_002746.3
p38	CCCGCTTATCTCATTAACAGGATG	AGGTCAGGCTTTTCCACTCAT	NM_001315.3
PI3K	GGGCGGGGTGAAGCTC	AGTCCTCTCACTGGCTTCCT	NM_181523.3
PKC	AGGACTGATGACCAAACACCC	CTCCTTTGCCACACACTTTGG	NM_002737.3
AMPK	AAGAATGGAAGGCTGGATGAAAAAG	GGGCCTGCATACAATCTTCCT	NM_006251.5
GSK3	CCGTCTCTCAACGCCATTCT	CTTCCCACGCCCTCTTGG	NM_019884.3
GAPDH	GTCAAGGCTGAGAACGGGAA	AAATGAGCCCCAGCCTTCTC	NM_002046.7
B-actin	GTGGCATCCACGAAACTACC	GTACTTGCGCTCAGGAGGAG	NM_001101.5

Supplementary Table 2 - antibodies

Antigen	Manufacturer	Cat. number	Application
c-kit	R&D Systems	AF1356	IHC
α-sarcomeric actin	Sigma	A2127	IHC, ICC
CD31	Abcam	ab182981	IHC
CD45	Abcam	ab10558	IHC
BrdU	Merck	11296736001	ICC
Ki67	BD Biosciences	BD556003	ICC
Smooth muscle actin	Sigma	A2547	ICC
von Willebrand factor	Dako	A0082	ICC
c-kit	R&D Systems	AF1356	Western blot
ß-actin	Cell Signaling	#3700	Western blot
Nkx2.5	GeneTex	GTX105711	Western blot
PECAM-1	R&D systems	AF806	Western blot
HGF	Santa Cruz	sc-374422	Western blot
PDGF-A	Santa Cruz	sc-9974	Western blot
EGF	GeneTex	GTX111176	Western blot
Wnt2	R&D Systems	AF3464	Western blot
Akt1	Cell Signaling	#2938	Western blot
Phospho-Akt1	Cell Signaling	#9018	Western blot
p38	Cell Signaling	#9212	Western blot
Phospho-p38	Cell Signaling	#9211	Western blot
Isotype control (PE-Vio)	Miltenyi	130-108-377	Flow cytometry
Isotype control (FITC)	Miltenyi	130-099-119	Flow cytometry
Anti-rabbit IgG Fluor-488	Life Technologies	10424752	Flow cytometry
VEGFR1	Enogene	E11-0594B	Flow cytometry
VEGFR2	Enogene	E02-1078	Flow cytometry
VEGFR3	Bioss	bs-1083R	Flow cytometry
PDGFRα-FITC	Miltenyi	130-115-336	Flow cytometry
PDGFRß-PE-Vio	Miltenyi	130-105-323	Flow cytometry
